# Chinese Society of Pediatric Anesthesiology Guideline for Pediatric Sedation (2025)

**DOI:** 10.1002/pan.70178

**Published:** 2026-04-10

**Authors:** Xingrong Song, Dongxu Lei, Yu Cui, Zhen Du, Yingping Jia, Yue Jin, Hang Tian, Ying Xu, Lifang Yang, Jianmin Zhang, Jijian Zheng, Yunxia Zuo, Liang Zhong, John Zhong, Maryrose Osazuwa, Elizabeth Drum, Charles Dean Kurth, Yue Huang, Qinghua Huang, Heqi Liu, Wei Liu, Rui Ma, Dongpi Wang, Tingting Wang, Zixin Wang, Siwei Wei, Lei Yang, Rui Zhou, Liumei Chen, Weifeng Yu, Jun Li, Mazhong Zhang, Li Zhang, Shuangquan Qu, Shoudong Pan, Rong Wei, Liming Cheng, Yaolong Chen, Xueping Li, Zhenyu Tang

**Affiliations:** ^1^ Department of Anesthesiology and Perioperative Medicine, Guangzhou Women and Children's Medical Center Guangzhou Medical University Guangzhou China; ^2^ Department of Anesthesiology Chengdu Women's and Children's Central Hospital Chengdu China; ^3^ Department of Anesthesiology The Affiliated Children's Hospital of Xiangya School of Medicine, Central South University (Hunan Children's Hospital) Changsha China; ^4^ Department of Anesthesiology Children's Hospital Affiliated of Zhengzhou University Zhengzhou China; ^5^ Department of Anesthesiology Children's Hospital, Zhejiang University School of Medicine Hangzhou China; ^6^ Department of Anesthesiology Children's Hospital of Chongqing Medical University Chongqing China; ^7^ Chevidence Lab Child & Adolescent Health Children's Hospital of Chongqing Medical University, National Clinical Research Center for Children and Adolescents' Health and Diseases, Ministry of Education Key Laboratory of Child Development and Disorders, Chongqing Key Laboratory of Child Neurodevelopment and Cognitive Disorders Chongqing China; ^8^ Department of Anesthesiology Affiliated Children's Hospital of Xi'an Jiaotong University Xi'an China; ^9^ Department of Anesthesiology, National Center for Children's Health Beijing Children's Hospital, Capital Medical University Beijing China; ^10^ Department of Anesthesiology Shanghai Children's Medical Center Affiliated to Shanghai Jiaotong University School of Medicine Shanghai China; ^11^ Department of Anesthesiology West China Hospital, Sichuan University Chengdu China; ^12^ Department of Anesthesiology Wuhan Children's Hospital (Wuhan Maternal and Child Healthcare Hospital) Tongji Medical College, Huazhong University of Science & Technology Wuhan China; ^13^ Department of Anesthesiology Oklahoma Children's Hospital Oklahoma City Oklahoma USA; ^14^ Department of Anesthesiology National Hospital Abuja Abuja Nigeria; ^15^ Department of Anesthesiology and Critical Care Medicine, Perelman School of Medicine, Children's Hospital of Philadelphia University of Pennsylvania Philadelphia Pennsylvania USA; ^16^ Department of Anesthesiology First Affiliated Hospital of Wenzhou Medical University Wenzhou China; ^17^ Department of Anesthesia and Perioperative Medicine The Second Affiliated Hospital and Yuying Children's Hospital of Wenzhou Medical University Wenzhou China; ^18^ Department of Anesthesiology Children's Hospital of Nanjing Medical University Nanjing China; ^19^ Department of Anesthesiology Capital Center for Children's Health, Capital Medical University Beijing China; ^20^ Department of Anesthesiology Shanghai Children's Hospital, Shanghai Jiao Tong University School of Medicine Shanghai China; ^21^ Department of Anesthesiology and Surgical Intensive Care Unit Kunming Children's Hospital Kunming China

**Keywords:** conscious sedation, deep sedation, hypnotics and sedatives, pediatrics, practice guideline

## Abstract

**Background:**

With the increasing variety of pediatric diagnostic procedures, a growing number of children require sedation for diagnostic examinations. Appropriate sedation protocols guarantee the safety of children during sedation and improve its efficiency. Currently, there are significant differences in pediatric sedation practices across countries, regions, and medical institutions.

**Methods:**

The Pediatric Anesthesiology Group of the Chinese Society of Anesthesiology organized experts from multiple countries to develop the “Chinese Society of Pediatric Anesthesiology Guideline for Pediatric sedation (2025)” based on evidence‐based medicine, considering the safety and efficacy, the preferences of children and parents, and drug accessibility.

**Results:**

The guideline provided 28 recommendations addressing 12 clinical issues related to pre‐sedation assessment, safety measures for sedation personnel and facilities, sedation protocols, sedation monitoring, and post‐sedation recovery.

**Conclusions:**

The guideline is planned to be disseminated globally through multiple channels, aiming to standardize the management of pediatric sedation and improve its safety and efficacy.

## 1 Background

There has been a rapid increase in pediatric diagnostic and therapeutic procedures over the past decades, such as Computed Tomography (CT), Magnetic Resonance Imaging (MRI), ultrasound, neurophysiological examinations, and ophthalmic examinations. However, due to the immature psychological development of children, their anxiety and fear issues about unfamiliar environments or medical procedures often lead to poor cooperation even in non‐invasive and painless examinations.

Additionally, some examinations require children to remain completely motionless for a long time to ensure imaging quality, which is extremely challenging for children. Consequently, most pediatric diagnostic examinations must be performed under sedation or even general anesthesia. Between 2020 and 2025, the number of tertiary hospitals in China performing over 10 000 pediatric sedation cases annually increased from 10 to 16, while those conducting over 1000 cases rose from 22 to 40 during the same period [1, 2]. Similarly, the Pediatric Sedation Research Consortium (PSRC) in the United States recorded over 400 000 pediatric sedation cases between 2007 and 2018, with numbers continuing to grow annually [3].

Levels of sedation were classified into mild sedation, moderate sedation, deep sedation, and general anesthesia based on the patient's response to verbal or painful stimuli, adequacy of airway, spontaneous ventilation, and cardiovascular function (Table 1) [4, 5]. Safe and effective sedation techniques can not only significantly alleviate children's anxiety and enhance their tolerance for medical procedures, but also reduce interruptions during diagnostic examination, avoid repeated exams, and minimize potential psychological harm. Compared with children undergoing surgery, those receiving sedation for diagnostic examination often have unclear diagnoses, more complex conditions, and a greater number of unknown factors, which increases the risk of serious complications, such as respiratory depression, hypoxemia, hypotension, and aspiration [6]. In low‐ and middle‐income countries, studies reported an overall adverse event rate of 24.4% during pediatric imaging sedation, including respiratory events (3.0%), cardiovascular events (0.7%), and prolonged sedation (15.8%) [7]. Developed countries reported a lower severe adverse event rate (1.75%), with airway obstruction (1.55%) being the most common complication [3].

**TABLE 1:** Continuum of depth of sedation: Definition of general anesthesia and levels of sedation.

	Mild sedation (anxiolysis)	Moderate sedation (conscious sedation)	Deep sedation	General anesthesia
Responsiveness	Normal response to verbal stimulation	Purposeful response to verbal or tactile stimulation	Purposeful response after repeated or painful stimulation	Unarousable, even with painful stimulus
Airway	Unaffected	No intervention required	Intervention may be required	Intervention often required
Spontaneous ventilation	Unaffected	Adequate	May be inadequate	Frequently inadequate
Cardiovascular function	Unaffected	Usually maintained	Usually maintained	May be impaired

Although extensive research has focused on methods and medications for pediatric sedation, there are still differences in clinical practice among different countries and regions, medical institutions, and professionals.

Regarding the sedation provider demographics, a Chinese questionnaire survey revealed that anesthesiologists are the primary evaluators (81.8%) and providers (53.4%) of pediatric sedation, followed by pediatric physicians, radiologists, and nurses [2]. Data from the PSRC in the United States showed pediatric intensivists (49.3%) and pediatric emergency physicians (28.2%) were primary sedation providers, while anesthesiologists accounted for only 9.8% [3, 8]. A survey across 19 African countries reported anesthesiologists (63%) were the main pediatric sedation providers, followed by trainees (29%) and nurses (13%) [9]. A European emergency department survey found that pediatricians or pediatric emergency physicians were the pediatric sedation providers in 70% and 80% of hospitals, respectively, and anesthesiologists were the providers in only 36% of hospitals [10]. In France, approximately 50% of pediatric CT/MRI sedations were performed by radiologists [11]. In Germany, the physician performing the examination is also responsible for sedation [12]. Regarding the prerequisite skills of providers, in China, 20% of hospitals require sedation providers to complete Pediatric Advanced Life Support (PALS) training and 28.4% of hospitals require sedation providers to have more than 5 years of pediatric anesthesia experience [2]. In Europe, 37% of institutions required PALS certification for all sedation providers [10]. A survey from Africa showed that only half of respondents received adequate training in pediatric sedation, and 84% of respondents were unaware of national pediatric sedation guidelines [9]. In South Africa, 82% of providers lacked formal pediatric sedation training, although the training program exists in this country [13]. Regarding the choice of sedatives, the most commonly used sedatives for children were chloral hydrate, dexmedetomidine, and propofol in China, with chloral hydrate usage declining while dexmedetomidine use is gradually increasing [1, 2]. In the United States, propofol was the predominant sedative, while pentobarbital (decreasing from 7.3% to 0.5%) and chloral hydrate (6.5% to < 0.1%) were declining, and dexmedetomidine use was steadily rising [3]. The most widely used pediatric sedative is chloral hydrate (87.7% of hospitals) in South Korean emergency departments [14], ketamine (92%) and benzodiazepines (82%) in Africa [9], midazolam (100%) and ketamine (90%) with a small amount of dexmedetomidine (10%) in European emergency departments [10], chloral hydrate (81.2%) and midazolam (72.6%) in the Netherlands [15], pentobarbital [11] in France and midazolam, ketamine/esketamine, propofol in Germany [12]. Regarding facilities for safety in sedation units, a Chinese survey reported that only 39.8% and 44.3% of hospitals had sedation rooms and recovery rooms respectively [2]. In South Korea, only 64.6% of emergency departments implemented patient monitoring during sedation and 20% of them had institutional pediatric sedation guidelines [14]. In Europe, merely 74% of emergency departments had safety and monitoring protocols for pediatric sedation [10]. Data from Germany reported that 5% of sedation units lacked oxygen supply and SpO_2_ monitoring, 25% of them lacked ECG and blood pressure monitoring equipment, and 15% lacked negative‐pressure suction devices [12]. In France, 35% of hospitals employed no monitoring, and fewer than 10% monitored blood pressure or capnography during pediatric CT/MRI sedation [11].

These findings demonstrated the critical challenges in pediatric sedation practice, including the lack of unified guiding principles, significant regional disparities in resources and training, and insufficient understanding and application of existing evidence. The sedation guidelines from the United States, South Korea, and Europe are primarily based on adult evidence, with limited provisions specific to pediatric sedation [5, 16, 17]. There were significant differences in physiology, pharmacology, and psychology between children and adults, which means adult‐derived evidence cannot be fully extrapolated to pediatric sedation. The pediatric sedation guidelines developed by the American Academy of Pediatrics (AAP) and the American Academy of Pediatric Dentistry (AAPD) covered invasive procedures such as wound debridement and suturing, fracture reduction, and dental treatments [18, 19]. These procedures require higher levels of analgesia but relatively lower levels of sedation. In contrast, diagnostic examinations are typically non‐invasive and involve no painful stimuli, eliminating the need for analgesia but demanding a deeper level of sedation to ensure complete immobility during prolonged examinations. Therefore, the AAP and AAPD sedation guideline may not be applicable to non‐invasive diagnostic examinations. In recent years, sedatives and protocols of pediatric sedation have evolved significantly, accompanied by emerging new research and evidence. A pediatric sedation guideline for diagnostic examinations based on the latest scientific evidence is needed to standardize the practice in this field.

The Pediatric Anesthesiology Group of the Chinese Society of Anesthesiology organized experts in pediatric anesthesia and evidence‐based medicine to establish a working group on pediatric sedation guidelines. The Working Group developed the recommendations by rigorously evaluating evidence, considering safety and efficacy, the preferences of children and parents, and drug accessibility, with the aim of standardizing pediatric sedation practices for diagnostic examinations.

## 2 Methodology

This guideline was developed in accordance with the 2014 WHO Handbook for Guideline Development [20], and references the Chinese Guiding Principles for Formulating/Revising Clinical Diagnosis and Treatment Guidelines (2022 Edition) [21]. The protocol and final document were prepared by applying the specific requirements of the Appraisal of Guidelines for Research and Evaluation (AGREE II), the Reporting Items for Practice Guidelines in Healthcare (RIGHT), and the Scientificity, Transparency, and Applicability Rankings (STAR) tool [22, 23]. The guideline was registered with the International Practice Guideline Registry (http://guidelines‐registry.org) under registration number PREPARE‐2024CN963, and the protocol is accessible through this platform.

### 2.1 Formation of the Guideline Development Working Group and Protocol

The Working Group was structured into the following sub‐units: Chief Experts, Steering Committee, Consensus Expert Panel, Secretariat Team, Evidence Evaluation Team, Methodology Team, and External Review Group. The detailed division of labor and the complete roster of members are available in Supporting Information S1.

### 2.2 Conflicts of Interest (COI) and Funding Sources

COI management for this guideline followed the Guidelines International Network (GIN) principles. All experts involved in the development and review completed and signed a Declaration of Interests (DOI) form, and no individuals with significant conflicts participated in any stage of guideline development. The Bethune Charitable Foundation provided funding for this guideline's development, covering expenses such as literature retrieval, evidence evaluation, publication fees, document printing, and post‐publication dissemination.

### 2.3 Intended Users and Target Populations

The expected users of the pediatric sedation guideline included all sedation providers (including anesthesiologists, non‐anesthesiologists, and nurses) who provide mild to deep pediatric sedation.

The target populations were children aged 0–18 years undergoing mild to deep sedation for non‐invasive diagnostic examination, excluding patients in Intensive Care Units (ICU) or those with mechanical ventilation.

### 2.4 Guidelines for Clinical Question Identification

Expert interviews were conducted with anesthesiologists, pediatricians, and pediatric sedation nurses, yielding 17 clinical questions related to pediatric sedation. The questions were structured according to the PICO framework (Population, Intervention, Control, Outcome). These questions were then developed into a questionnaire and subjected to a Delphi survey among experts, ultimately identifying 12 key clinical questions to be addressed in this guideline (Supporting Information S2).

### 2.5 Literature Search and Study Selection

Based on the predefined clinical questions, we comprehensively searched the following databases: PubMed, Embase, Cochrane Library, Web of Science, CNKI (China National Knowledge Infrastructure), Wanfang Data, and SinoMed (Chinese Biomedical Literature Database). The search was restricted to publications in Chinese and English, with a cutoff date of October 1, 2024. A combined approach of Medical Subject Headings (MeSH) and free‐text terms was employed (Supporting Information S3). For example, regarding pediatric sedative selection, the key terms included: “Child,” “neonat*,” “Infant,” “Child, Preschool”, “Adolescent”, “procedural sedation”, “Hypnotics and Sedatives”, “moderate sedation”, “deep sedation”, “Dexmedetomidine”, “chloral hydrate”, “Midazolam”, “Phenobarbital”, “Sevoflurane”, “Nitrous Oxide”, “propofol”. Inclusion criteria comprised systematic reviews (SR), meta‐analysis, network meta‐analysis, randomized controlled trials (RCTs), cohort studies, and case series studies focusing on pediatric sedation. Exclusion criteria eliminated studies with irrelevant topics, gastrointestinal endoscopy, interventional surgery, invasive procedures, non‐pediatric populations, patients in ICU, unavailable full texts, or non‐Chinese/English publications. For each clinical question, two evidence evaluation team members independently screened literature by title, abstract, and full text, followed by cross‐verification; discrepancies were resolved through group discussion or methodology team consultation.

### 2.6 Determining the Level of Evidence and Recommendation Level

The AMSTAR II tool was employed to assess the risk of bias in meta‐analysis, the Cochrane Risk of Bias 1 (ROB1) tool for RCTs, and the Newcastle‐Ottawa Scale (NOS) for case–control and cohort studies. The determination of evidence levels and recommendation level was primarily based on the Oxford Centre for Evidence‐Based Medicine (CEBM) classification system (Table 2) [24], with clinical practical needs also being taken into consideration. Two members of the Evidence Evaluation Group independently conducted these evaluations and graded, and any discrepancies were resolved through discussion or consultation with the Methodology Group.

**TABLE 2:** Oxford Centre for Evidence‐Based Medicine: Levels of evidence (March 2009).

Grades of recommendation	Level of evidence	Evidence
A	1a	SR (with homogeneity) of RCTs
	1b	Individual RCT (with narrow Confidence Interval)
	11c	All or none case series study
B	2a	SR (with homogeneity) of cohort studies
	2b	Individual cohort study (including low quality RCT; for example < 80% follow‐up)
	2c	“Outcomes” research; Ecological studies
	3a	SR (with homogeneity) of case–control studies
	3b	Individual case–control study
C	4	Case‐series (and poor quality cohort and case–control studies)
D	5	Expert opinion without explicit critical appraisal, or based on physiology, bench research or “first principles”

### 2.7 Formulation of Recommendations

The Evidence Evaluation Group and Secretariat developed preliminary recommendation decision tables based on synthesized evidence from clinical studies and consideration of implementability. Two rounds of Delphi surveys were conducted between March 28 and August 5, 2025, and a consensus rate of over 75% is required to establish expert consensus (Supporting Information S4).

### 2.8 Manuscript Development, Revision, and External Review

The Secretariat completed the initial draft of these guidelines following the RIGHT reporting standards. After iterative discussions and modifications by the Steering Committee, Consensus Expert Group, and Evidence Evaluation Group, the draft underwent independent appraisal by three external reviewers.

### 2.9 Guideline Release, Dissemination, and Updates

The guideline was planned to be disseminated globally through academic journals, online and offline academic conferences, and official public accounts to facilitate broader adoption. The guidelines will be updated every 3 to 5 years based on evolving evidence‐based research and relevant policy changes, with the aim of further standardizing the practice of pediatric sedation.

## 3 Clinical Question

Through two rounds of the Delphi method, the consensus expert group reached consensus on 31 recommended opinions for 12 clinical questions and merged some of them, ultimately determining 28 recommended opinions.

### 3.1 Clinical Question 1: What Assessments Are Required Prior to Pediatric Sedation?

#### 3.1.1 Recommendation

1.1 It is recommended to evaluate the child's upper respiratory infection (URI) status prior to pediatric sedation (Recommendation Level: B; Level of evidence: 2b, Consensus rate 100% (16/16)).
1.2 It is recommended to assess the child's airway patency prior to pediatric sedation to determine the need for artificial airway support (Recommendation Level: C; Level of evidence: 4; Consensus rate 93.8% (15/16)).
1.3 It is recommended to evaluate the child's developmental status prior to pediatric sedation (Recommendation Level: B; Level of evidence: 2b, Consensus rate 100% (16/16)).

#### 3.1.2 Rationale for Recommendation

In addition to routine assessments (including height, weight, heart rate, respiratory rate, oxygen saturation, American Society of Anesthesiologists (ASA) classification, current/past medical history, and medication history), special attention should be directed to upper respiratory infection (URI) status, airway patency, and developmental abnormalities (e.g., intellectual disability, autism spectrum disorder, attention deficit disorder) [4]. The presence of URI is strongly associated with an increased incidence of post‐sedation airway adverse events and higher demand for airway interventions [25]. Thus, pre‐sedation URI assessment is essential. Children requiring head/neck MRI often exhibit varying degrees of impaired consciousness or airway‐compromising conditions, which elevate their risk of respiratory complications during sedation [26]. A thorough pre‐sedation airway evaluation is critical to guide decisions on artificial airway support.

Although existing evidence does not conclusively demonstrate reduced sedation success rates in children with developmental disorders [27], this population generally demonstrates lower treatment compliance and requires prolonged immobility during MRI. Systematic pre‐sedation developmental assessment is therefore advised to tailor individualized anesthesia management.

#### 3.1.3 Evidence Summary

A cohort study of 83 491 sedated children demonstrated that upper respiratory infection (URI) status significantly influenced the incidence of airway adverse events. Among children with no recent URI, the incidence of airway‐related complications was 7.9% (5560/70 830). In contrast, those with a recent URI exhibited a higher incidence of 10.4% (348/3354). Further stratification revealed that children with URI accompanied by clear secretions had a complication rate of 16.0% (1381/9307), while those with thick secretions experienced the highest incidence at 24.0% (158/658). Although severe adverse events—including laryngospasm, aspiration, unplanned intubation, and hospitalization—occurred in < 1% of cases overall, laryngospasm rates varied significantly across groups (*p* < 0.001): 0.3% in the no‐URI group, 0.5% in the recent‐URI group, 0.7% in the URI‐with‐clear‐secretions group, and 0.9% in the URI‐with‐thick‐secretions group [25].

A retrospective cross‐sectional study of 82 infants (0–12 months) undergoing head/neck MRI under sedation with spontaneous respiration reported that 35% of these cases developed airway obstruction, and 83% were associated with reduced consciousness levels [26].

A retrospective cohort study of 191 children undergoing MRI sedation reported an overall success rate of 92.7%. Children with developmental disorders showed reduced sedation success (OR = 0.215, 95% CI: 0.060–0.774). However, after adjusting for age, developmental disorders were not independently associated with sedation failure, leaving the impact of developmental status inconclusive [27].

### 3.2 Clinical Question 2: Should an Anesthesiologist Be Involved in Pediatric Sedation?

#### 3.2.1 Recommendation

2.1 It is recommended that anesthesiologists be involved in pediatric sedation (Recommendation level: B; Level of evidence: 2b, Consensus rate 93.8% (15/16)).
2.2 It is recommended that, for sedation regimens using a single agent (dexmedetomidine or midazolam) in ASA class ≤ II children, sedation can be administered by either an anesthesiologist or a qualified non‐anesthesiologist physician or nurse (Recommendation Level C; Level of evidence 4, Consensus rate 87.5% (14/16)).

#### 3.2.2 Rationale for Recommendation

In recent years, data from various sources including PSRC have shown that pediatric sedation can be safely and effectively administered by appropriately trained non‐anesthesiologists with an adverse event rate of approximately 10% [28]. According to a PSRC series report, about 9.5%–10.8% of sedation cases were performed by anesthesiologists from 2007 to 2018. Another report found that only 12% of pediatric sedation in South Korea was performed by anesthesiologists [29]. In Japan, the proportion of pediatric sedation performed by anesthesiologists is also extremely low [30]. Different from other countries, pediatric sedation in China was performed primarily by anesthesiologists and secondly by nurses [2]. It has also been reported that nurse‐led sedation can achieve satisfactory efficacy and safety in children abroad. These nurses are typically trained and qualified in pediatric sedation, and their cases are limited to procedures using a single sedative drug and involving children classified as ASA ≤ II [31, 32, 33]. Furthermore, anesthesiologists are always available to provide rescue for sedation failure and to perform sedation for critically ill children. Although many studies have reported successful pediatric sedation by non‐anesthesiologists, the success rate of sedation for imaging studies performed by non‐anesthesiologists varies widely, with failure rates ranging from 2% to 15%, and sedation provided by non‐anesthesiologists is associated with higher rates of adverse events in high‐risk populations [2]. Safety remains the most important concern when sedation is performed by non‐anesthesiologists. Two cohort studies have shown that the participation of anesthesiologists in pediatric sedation can effectively reduce the incidence of adverse events and is more cost‐effective than that of non‐anesthesiologists [34, 35]. Therefore, it is recommended that anesthesiologists should be involved in the process of pediatric sedation. Currently, there is limited research on pediatric sedation performed by non‐anesthesiologists such as emergency physicians, intensivists, and radiologists. However, given the educational background and clinical experience, we conclude that non‐anesthesiologists with adequate training can perform sedation effectively and safely in children with ASA ≤ II and receiving a single‐agent sedation regimen.

The Chinese expert consensus on anesthesia/sedation for pediatric procedures suggested that the anesthesia/sedation process should be supervised by at least one anesthesiologist qualified as an attending physician with more than 1 year of pediatric anesthesia experience [4]. Additionally, each sedation unit should be staffed with at least one medical staff trained in anesthesia/sedation and PALS to assist the anesthesiologist. The clinical practice statement from the European Society of Pediatric Anesthesia also emphasizes the need for teamwork in pediatric sedation and emphasizes that sedation should be provided by a pediatric anesthesiologist or intensivist for high‐risk patients (ASA III and IV) [36]. The expert consensus developed by the Italian Society for the Sciences of Anesthesia, Analgesia, Resuscitation and Critical Care states that to ensure the effectiveness and safety of pediatric sedation, anesthesiologists or intensivists with pediatric experience are needed [37].

#### 3.2.3 Evidence Summary

A retrospective cohort study of 13 072 children showed that the type of sedation provider can influence the incidence of adverse events when dexmedetomidine was used as the preferred pediatric sedative34. The overall incidence of adverse events was 3.6% (95% CI: 3.3%–3.9%). The highest incidence of adverse reactions during sedation was observed among emergency physicians, which was 7.82%, followed by intensivists 5.83%, other medical staff 4.14%, residents 3.55%, and anesthesiologists 1.31%. After adjusting for factors such as weight, prematurity, and ASA status, the adjusted odds ratios for adverse events during sedation performed by emergency physicians, intensivists, other medical staff, and residents when compared to anesthesiologists were 8.5 (*p* < 0.001), 5.3 (*p* < 0.001), 4.6 (*p* < 0.001), and 3.7 (*p* = 0.007), respectively.

A single‐center observational study included 1546 children requiring MRI to evaluate the cost‐effectiveness of sedation by anesthesiologists versus non‐anesthesiologists [35]. The results showed that the failure rate of sedation by non‐anesthesiologists was 17.5%, with no failure by anesthesiologists. A cost‐effectiveness analysis with sedation success as the outcome showed that the average cost per patient for sedation by anesthesiologists was US $84.2, followed by US $74.2 to US $92.7 for intravenous sedation by non‐anesthesiologists, US $112.1 to US $458.3 for oral or rectal sedation by non‐anesthesiologists, and US $605.4 for general anesthesia by anesthesiologists. Under Japanese health care cost standards, sedation administered by an anesthesiologist was more cost‐effective than by a non‐anesthesiologist, who had a higher rate of failure for children undergoing MRI.

A single center audit included 772 children, classified as ASA grade I‐II, full‐term, and < 18 years, to evaluate the efficacy and safety of intranasal dexmedetomidine administered by a nurse‐led sedation team for pediatric sedation [31]. The results showed a sedation success rate of 91%, 7.8% of children required additional intervention, 4.8% required an additional dose of intranasal dexmedetomidine, and 1.8% received additional sedatives. Sedation failure occurred in 1.6% of cases. The incidence of adverse events was 1.7%, with no serious adverse events reported.

A retrospective case study evaluated nurse‐led pediatric sedation over a 7‐year period, a uniform assessment was performed prior to sedation, and children with an ASA ≥ 2 required a secondary evaluation by an anesthesiologist to determine their suitability for a nurse‐led sedation protocol [33]. The study included 3151 children, and 2293 (72.7%) received sedation. The main sedative drugs used were dexmedetomidine or midazolam, and the success rate of sedation was 99.7%. No serious adverse events occurred, and 37 (1.6%) patients experienced adverse events requiring intervention.

Another retrospective quality improvement study evaluated 6 years of nurse‐led sedation with dexmedetomidine for pediatric MRI [38]. A total of 1987 nurse‐administered MRI sedation cases were included, and all cases were professionally assessed to determine their suitability for nurse‐led sedation. In the intravenous medication group (*n* = 1768), the sedation success rate was 98.9% and 95.0% in the intravenous medication group and intranasal medication group, with an adverse event incidence of 3.8% and 1.8%, respectively.

### 3.3 Clinical Question 3: Which Competency Should Pediatric Sedation Provider Possess?

#### 3.3.1 Recommendation

3.1 Competency of PBLS is recommended for all pediatric sedation providers, and competency of advanced training in pediatric life support should be required for those performing deep sedation (Recommendation Level: A, Level of evidence: 5, Consensus rate 100% (16/16)).
3.2 It is recommended that providers administering sedation to children under 6 years or at high risk should either hold the qualification of an attending physician (or higher) or have at least one year of pediatric anesthesia experience (Recommendation Level: A, Level of evidence: 5, Consensus rate 100% (16/16)).
3.3 It is recommended structured training in sedation (including assessment of first‐aid simulation) for all pediatric sedation providers (Recommendation Level: A, Level of evidence: 5, Consensus rate 100% (16/16)).

#### 3.3.2 Rationale for Recommendation

PBLS is a standardized competency required for cardiopulmonary resuscitation and emergency care. It directly improves the odds of successful resuscitation and reduces the mortality rate of cardiac arrest. Specialized emergency skills are required for pediatric patients due to the physiological characteristics of children. The certification of PALS can effectively reduce the incidence of severe respiratory and cardiovascular complications in pediatrics [37, 39, 40, 41, 42]. In the Chinese expert consensus on anesthesia/sedation for pediatric procedures outside the operating room, the ASA, AAP, AAPD Sedation Guidelines, the National Institute for Health and Clinical Excellence (NICE) guidelines in the UK, and the ITALIAN scientific society of anesthesia, analgesia, resuscitation and intensive care (SIAARTI) expert consensus, PBLS is considered an essential prerequisite for pediatric sedation providers, while PALS is required for deep sedation [4, 37, 39, 40, 41, 42]. Although lacking high‐quality evidence, the requirement for PBLS and PALS proficiency has formed a consensus in clinical practice worldwide. Therefore, our expert panel strongly recommends that pediatric patient sedation practitioners should be qualified with PBLS and receive advanced training in pediatric life support.

There are significant differences in physiology and anatomy between children and adults. Children ≤ 6 years (especially those ≤ 6 months) may be at the highest risk of adverse events [43]. They are particularly susceptible to the effects of sedatives on respiratory movements, airway patency, and protective airway reflexes [44]. A cross‐sectional study indicated that most hospitals in China believed that pediatric sedation providers should have the qualification of attending physician and at least 5 years of pediatric anesthesia experience [2]. The Chinese expert consensus on anesthesia/sedation for pediatric procedures outside the operating room (2017) also recommended that physicians performing pediatric sedation or anesthesia should have the qualification of attending physician or above, and have at least 1 year of pediatric anesthesia experience [4]. Based on the current clinical situation and relevant experts' consensus, our expert group strongly recommended that moderate‐to‐deep sedation for children under 6 years of age or high‐risk pediatric patients should be performed by senior physicians with extensive experience in pediatric sedation or anesthesia to ensure patient safety. For children over 6 years old without significant risk factors, sedation may be implemented by experienced residents who have received comparable training.

At present, due to the shortage of anesthesiologists, non‐anesthesiologists often perform sedation on children in dentistry and radiology [28, 45]. They may be comparably unsubstantial in airway management and emergency resuscitation. Therefore, pediatric sedation providers are recommended to have professional knowledge and skills in risk assessment and sedation implementation, and the competency to identify and handle all adverse events that may occur [28, 46, 47]. According to the experts consensus in China, in addition to PALS, medical staff also need to receive a structured “anesthesia/sedation training”. The AAP and AAPD sedation guidelines recommend that during deep sedation, there should be a supervisor who has received PALS and airway management training [39, 40]. Therefore, our expert group strongly recommended that pediatric sedation providers should receive structured training in sedation.

#### 3.3.3 Evidence Summary

At present, no direct evidence has been found regarding the qualifications of pediatric sedation providers.

### 3.4 Clinical Question 4: What Conditions Should Be in Place for a Pediatric Sedation Facility?

#### 3.4.1 Recommendation

4.1 It is recommended that all healthcare facilities providing pediatric sedation should be equipped with dedicated sedation rooms and post‐sedation recovery rooms (Recommendation Level: A, Level of evidence: 5, Consensus rate 100% (16/16)).
4.2 It is recommended that pediatric sedation facilities should be equipped with emergency equipment, including airway management supplies: oxygen supply devices with flow regulators (at least two independent oxygen sources), anesthesia machines or ventilators, manual resuscitators, nasal cannulas and face masks, oropharyngeal or nasopharyngeal airways, supraglottic airway devices (e.g., laryngeal masks), laryngoscopes, endotracheal tubes with stylets, and suction devices, defibrillators, emergency medications, cardiopulmonary resuscitation (CPR) equipment, and communication devices (Recommendation Level: A, Level of evidence: 5, Consensus rate 100% (16/16)).
4.3 It is recommended that the pediatric sedation areas should be equipped with basic monitoring devices, including pulse oximetry, electrocardiogram (ECG), blood pressure monitor, and stethoscope; and capnography is suggested (Recommendation Level: A, Level of evidence: 5, Consensus rate 100% (16/16)).

#### 3.4.2 Rationale for Recommendation

Any level of sedation may progress to deep sedation or even anesthesia, requiring dedicated facilities to monitor and observe these sedated children just as in anesthesia management, in order to promptly detect and address potential risks until the sedation is finished. Establishing a unified sedation room and post‐sedation recovery rooms allows for the centralized monitoring of children, facilitating better care and supervision. A nationwide survey from China shows that the nationwide proportion of hospitals with dedicated sedation and recovery rooms was not exceeding 50% [2]. But it has significantly increased compared to 5 years ago, with a higher percentage of 81.3% and 75% in hospitals performing over 10 000 sedations annually. The guidelines from Korea and Europe recommend that dedicated sedation rooms and post‐sedation recovery rooms are necessary for children or adult [16, 17].

Pediatric sedation carries potential risks (such as hypoxia, apnea, bradycardia, hypotension, and vomiting), even more unpredictable than anesthesia risks due to the presence of unclear diagnoses. Therefore, all facilities providing sedation should be equipped with easily accessible and standard resources to manage these risks and ensure child safety [1, 16, 48, 49]. The sedation guidelines or expert consensus from China, the United States and Korea strongly advocate for sedation to be administered in settings equipped with monitoring devices, emergency medications, airway management equipment, and resuscitation resources. and suggested establishing standardized environments with the minimum essential medications and equipment required for sedation to optimize clinical management and reduce the incidence of sedation‐related complications [4, 16, 19].

No high‐quality direct evidence is currently available for this clinical issue. However, given the critical importance of a dedicated environment with necessary emergency and monitoring devices to ensure the safety of sedated children, the expert group unanimously endorses this recommendation with an upgraded level of Grade A.

#### 3.4.3 Evidence Summary

There is currently no direct evidence available regarding the conditions for pediatric sedation settings.

A 2020 cross‐sectional survey in China revealed that among 58 hospitals providing moderate‐to‐deep pediatric sedation, only 24.1% and 32.8% of them had dedicated sedation rooms and post‐sedation recovery rooms, respectively 1. And the proportion rose to 39.8% and 44.3% in recent research published in 20 242. Both studies indicate that establishing dedicated areas for providing sedation services facilitates gathering the children to get appropriate monitoring and care in one place. and recommended that all healthcare facilities offering pediatric sedation procedures should be equipped with dedicated sedation rooms and post‐sedation recovery rooms.

A 2016 meta‐analysis investigating the incidence of adverse events during procedural sedation in pediatric emergency departments reported the following most common complications: vomiting (5.55%, 95% CI: 4.52%–6.58%), agitation (1.79%, 95% CI: 1.22%–2.37%), hypoxemia (1.48%, 95% CI: 1.02%–1.93%), and apnea (0.71%, 95% CI: 0.32%–1.10%) [48]. A 2017 prospective multicenter observational cohort study including 9657 children on risk factors of adverse events in pediatric emergency sedation identified that the total adverse events rate was 11.7% (95% CI: 0.64%–1.69%), with hypoxemia (5.6%) and vomiting (5.2%) being the most frequent. Severe adverse events rate was 1.1% (95% CI: 0.5%–1.7%), and the rate of children requiring critical interventions was 1.4% (95% CI: 0.7%–2.1%) [49].

A 2024 retrospective cohort study including 3149 outpatient children receiving intranasal dexmedetomidine combined with oral midazolam for sedation reported a 4.70% incidence of serious adverse events, including bradycardia (2.03%), nausea/vomiting (0.98%), hypotension (0.89%), hypertension (0.57%), somnolence (0.10%), irritability (0.10%), and hypoxemia (0.03%) [50]. A 2019 retrospective cohort study of 17 948 outpatient children receiving intranasal dexmedetomidine‐ketamine combination for sedation reported 0.59% (95% CI: 0.48–0.71) of mild adverse events, including nausea/vomiting (0.3%, 95% CI: 0.22–0.39), oxygen desaturation (0.22%, 95% CI: 0.16–0.30), upper airway obstruction (0.11%, 95% CI: 0.07–0.17), emergence delay (0.07%, 95% CI: 0.03–0.12), and clinically significant heart rate/blood pressure fluctuations requiring intervention (0.02%, 95% CI: 0.21–0.37), with 4 severe adverse events (3 upper airway obstruction and 1 arrhythmia) [51].

A 2021 prospective observational study on propofol‐associated adverse events during pediatric sedation documented sedation‐related respiratory complications in 3.9% of 2569 children [52].

### 3.5 Clinical Question 5: What Contingency Plans Should Be Established for Pediatric Sedation?

#### 3.5.1 Recommendation

5.1 It is recommended to establish contingency protocols for peri‐sedation nausea, vomiting and reflux‐related aspiration, regardless of fasting status, to prevent severe adverse events (Recommendation Level: C, Level of evidence: 4, Consensus rate 100% (16/16)).
5.2 It is recommended that contingency plans should be developed for airway impairment or respiratory suppression (e.g., laryngospasm, endotracheal intubation, cricothyroid membrane puncture), as well as for hypoxia‐induced hypotension, cardiac arrest, and other critical adverse events, particularly in high‐risk pediatric patients (Recommendation Level: A, Level of evidence: 1a, Consensus rate 100% (16/16)).
5.3 It is recommended that continuous monitoring of sedation depth is advised, with contingency plans addressing both inadequate sedation and oversedation (Recommendation Level: B, Level of evidence: 2b, Consensus rate 100% (16/16)).

#### 3.5.2 Rationale for Recommendation

Sedation‐related adverse events commonly include nausea/vomiting, respiratory depression, agitation, and unintended sedation depth (over‐sedation or under‐sedation), while severe complications may encompass aspiration, laryngospasm, unplanned intubation, unplanned hospitalization, cardiac arrest, or death [53, 54]. High‐risk factors include younger age [55], coexisting respiratory/neurological disorders, and congenital heart disease [53]. Notably, nausea/vomiting represents the most frequent peri‐sedation adverse event, with fasting status for liquids/solids not being an independent risk factor for emesis or aspiration [54] in all children. No matter fasting or not, emergency plans for nausea, vomiting, and reflux aspiration should be in place.

Respiratory depression may progress to hypoxia‐related critical events such as hypotension and cardiac arrest, necessitating contingency plans. Respiratory monitoring should be implemented, with interventions including bag‐mask ventilation, oropharyngeal airway placement, or positive pressure ventilation as needed. Advanced airway management (e.g., endotracheal intubation or cricothyroid membrane puncture) must be prepared. Risk factors for unplanned intubation include ASA class III, prematurity, gastroesophageal reflux disease, or congenital heart disease [56]. High‐risk populations require prioritized airway management protocols and artificial airway establishment preparedness.

Significant variability exists in sedation depth, with substantial proportions of pediatric patients failing to achieve target sedation levels, experiencing over‐sedation, or deviating from intended sedation planes [57]. Sedation providers must demonstrate competency in depth recognition and maintain equipment and skills for implementing sedation depth adjustment protocols or cardiopulmonary support when required [19].

#### 3.5.3 Evidence Summary

A retrospective cross‐sectional study of 5800 pediatric patients requiring sedation in MRI and special inspection departments reported an overall incidence of sedation‐related adverse events of 2.72%, with a mortality rate of 0.03%. Common adverse events included nausea and vomiting (0.78%) and reflux with aspiration (0.52%) [53]. A case–control analysis of 139 142 procedural sedation cases identified 75 serious adverse events (aspiration, death, cardiac arrest, or unplanned hospitalization), with aspiration incidence at 0.097‰ in fasted versus 0.079‰ in non‐fasted patients (OR = 0.81; 95% CI: 0.08–4.08; *p* = 0.79) [54].

A systematic review of 41 studies involving 13 883 procedural sedations found during sedation the incidence of hypoxia was 1.48‰ (95% CI: 10.2–19.3), apnea was 7.1‰ (95% CI: 3.2–11.0), and 5.0‰ (95% CI: 2.3–7.6) of cases required bag‐mask/oropharyngeal airway interventions, with severe respiratory adverse events including laryngospasm 2.9‰ (95% CI: 1.1–4.7) and unplanned intubation 0.4‰ (95% CI: 0–0.8) [48]. A cross‐sectional study of 1000 neonates receiving 10% chloral hydrate for sedation reported apnea incidence of 0.5%, significantly higher in preterm (0.4%) versus term neonates (0.1%) (*p* < 0.05) [55]. A single‐center retrospective study including 1165 pediatric MRI sedation demonstrated an unplanned intubation rate of 2% (95% CI: 0.0123–0.0295), and multivariate analysis identified risk factors were ASA class III (OR = 1.212, *p* < 0.001), prematurity (OR = 2.317, *p* < 0.001), gastroesophageal reflux disease (OR = 1.474, *p* < 0.001), and congenital heart disease (OR = 1.118, *p* < 0.001) [56].

A prospective cohort study of 86 children undergoing moderate to deep sedation found only 53% (BIS‐monitored) or 72% (UMSS scale) children achieved target depth, with failure rates ranging from 12% to 28%, and 8% experienced oxygen desaturation or airway events during deep sedation [57].

### 3.6 Clinical Question 6: What Is the Optimal Sedation Regimen for Pediatric Patients?

#### 3.6.1 Recommendation

6.1 It is recommended oral midazolam for pediatric mild sedation (Recommendation level: A, Level of evidence: 1b, Consensus rate 93.8% (15/16)).
6.2 It is recommended dexmedetomidine for pediatric moderate sedation (Recommendation level: A, Level of evidence: 1a, Consensus rate 87.5% (14/16)).
6.3 It is recommended sevoflurane or combination regimens for pediatric deep sedation (Recommendation level: A, Level of evidence: 1a, Consensus rate 87.5% (14/16)).

#### 3.6.2 Rationale for Recommendation

The ideal sedative medication should meet the following characteristics, including easy administration, high success rate, rapid onset, controllable duration of action, quick metabolism without accumulation, and minimal adverse effects. Besides, the route of administration and the type of examinations must also be considered when selecting a sedative.

Oral and intranasal administration are noninvasive methods, which are simple to perform without additional pain and well accepted by children without intravenous (IV) access and their caregivers. IV administration provides rapid onset and a high success rate for sedation. However, it carries a higher risk of adverse events, such as respiratory depression and hypotension, requiring closer monitoring during sedation.

Different diagnostic examinations require varying levels of sedation depth. For examinations such as cardiac ultrasound and non‐contrast CT scans, which only necessitate mild to moderate sedation, a single sedative agent is usually sufficient to achieve satisfactory sedation. However, for prolonged or highly stimulating examinations requiring deep sedation such as MRI or in children with neurological disorders, a single‐agent regimen often fails to provide adequate sedation success rates. In such cases, combination of different sedation may enhance efficacy through synergistic effects without increasing adverse events [58, 59].

Dexmedetomidine, as a highly selective α2‐adrenergic receptor agonist, induces a sleep‐like sedation state, demonstrating superior efficacy compared to chloral hydrate and midazolam, with lower risks of respiratory depression and agitation [60, 61, 62]. However, it may cause bradycardia without requiring clinical intervention. Dexmedetomidine is primarily administered intranasally for pediatric sedation. Intranasal dexmedetomidine is absorbed either through the nasal mucosa or via the olfactory pathway, bypassing hepatic first‐pass metabolism, making it widely used in pediatric sedation. The recommended dose is 2–3 μg/kg. Compared to IV administration, intranasal delivery provides prolonged sedation duration and more stable plasma concentrations [63, 64].

Chloral hydrate is a traditional sedative which has a satisfactory sedative effect, but with respiratory depression risk at high dosage [65, 66]. It is primarily administered orally or rectally. Oral use is limited by bitter taste, vomiting, and low bioavailability due to first‐pass metabolism [61]. Rectal administration improves sedation success but may cause discomfort, resistance, and increased risk of rectal mucosal irritation or infection [67, 68]. Given its lower sedation success rate compared to dexmedetomidine and higher risk of adverse events, chloral hydrate is not recommended as a first‐line agent for moderate‐to‐deep sedation. However, compared to dexmedetomidine, chloral hydrate is more readily available and lower in price. Therefore, it can be used as an alternative for moderate sedation in settings where dexmedetomidine is unavailable or its use is limited by cost constraints.

Midazolam is the most used benzodiazepine in pediatric sedation, which can be administered intravenously (IV), orally, or intranasally. IV midazolam provides rapid onset and effective sedation but carries higher risks of agitation and respiratory depression [69, 70]. The sedative effects of oral and nasal administration of midazolam are comparable. However, intranasal administration may cause significant mucosal irritation. while the oral formulation has a sweet taste and is more readily accepted by children. Therefore, the oral route is recommended as first‐line administration. Midazolam provides less effective sedation compared to dexmedetomidine and chloral hydrate [69, 70, 71]. Therefore, it is only recommended for pre‐operative sedation or examinations requiring mild sedation such as premedication or echocardiography when used alone.

Combination of oral midazolam and intranasal dexmedetomidine enhances sedation success, making it suitable for procedures requiring deeper sedation such as MRI [72].

Sevoflurane and propofol are short‐acting general anesthetics that can induce deep sedation or general anesthesia at therapeutic doses. Both agents offer rapid onset, quick recovery, and excellent controllability, and are widely used for pediatric MRI sedation [73, 74]. Sevoflurane is associated with a higher incidence of respiratory adverse events (e.g., respiratory depression, airway obstruction) and emergence agitation. Its administration requires specialized equipment such as anesthesia machine and vaporizer. It may necessitate supraglottic airway devices such as laryngeal mask for ventilation support in cases of respiratory compromise. Thus, sevoflurane sedation should only be administered by anesthesiologists [75, 76]. Propofol, when used alone for deep sedation, often requires higher doses, increasing the risk of respiratory depression and hypotension [43]. Consequently, the American Guidelines for Sedation by Non‐Anesthesiologists recommended avoiding propofol for routine sedation. Recent studies suggest that combining propofol with dexmedetomidine or ketamine reduces propofol dosage and associated adverse events during MRI sedation [77, 78]. Therefore, propofol is not recommended to be used as a sole agent for pediatric sedation but could be considered in combination regimens for deep sedation.

#### 3.6.3 Evidence Summary

##### 3.6.3.1 Midazolam

One meta‐analysis demonstrates superior sedation efficacy of intranasal midazolam compared to intranasal ketamine for premedication. One RCT found no difference in sedation effectiveness among intranasal midazolam, intranasal ketamine, and oral chloral hydrate for echocardiographic sedation, though intranasal midazolam showed a faster onset time. One meta‐analysis indicates comparable sedation effects between oral midazolam and intranasal midazolam for premedication.

A 2022 meta‐analysis of 16 RCTs involving 1066 children demonstrated that intranasal midazolam showed higher sedation satisfaction rates compared to intranasal ketamine (61.76% vs. 40.74%, RR = 1.53, 95% CI: 1.28–1.83, *I* ^2^ = 26%), faster onset (SMD = −0.59, 95% CI: −0.90 to −0.28, *I* ^2^ = 0%), and shorter recovery time (SMD = −1.06, 95% CI: −1.83 to −0.28, *I* ^2^ = 82%) for pediatric premedication [79].

A 2017 RCT including 217 cases found the success rates of intranasal midazolam (0.2 mg/kg), intranasal ketamine (4 mg/kg) and oral chloral hydrate (50 mg/kg) were comparable (95.9% vs. 95.9% vs. 94.5%, *p* > 0.05) in pediatric echocardiographic sedation. But the onset time of midazolam was shorter than both ketamine and chloral hydrate (14 min [IQR: 7–65 min] vs. 34 min [12–56 min] vs. 40 min [25–57 min], *p* < 0.001) [58].

A 2020 meta‐analysis of 14 RCTs involving a total of 901 children. The results showed that for preoperative sedation in children, there was no significant difference in the sedative effects between oral midazolam and intranasal midazolam during anesthesia induction with mask acceptance (RR = 1.02, 95% CI: 0.93–1.13, *p* = 0.64, *I* ^2^ = 0%) and parental separation (RR = 0.99, 95% CI: 0.89–1.10, *p* = 0.90, *I* ^2^ = 53%) [80].

##### 3.6.3.2 Dexmedetomidine

Three meta‐analyses consistently demonstrate superior sedation success rates with dexmedetomidine compared to oral chloral hydrate for pediatric sedation. Two meta‐analyses indicate dexmedetomidine achieves higher sedation success rates than oral midazolam in various pediatric sedation scenarios (including both procedural sedation and premedication). One meta‐analysis shows intranasal dexmedetomidine provides higher sedation success rates than intranasal ketamine for both procedural sedation and premedication in children. One meta‐analysis reveals comparable sedation effects between intravenous dexmedetomidine and propofol during MRI examinations, and a lower incidence of hypoxia for dexmedetomidine.

A 2022 systematic review of 14 RCTs involving 3749 children showed that intranasal dexmedetomidine achieved higher sedation success rates (RR = 1.139, 95% CI: 1.051–1.235, *I* ^2^ = 86.7%, *p* < 0.001), faster onset of sedation (WMD = −0.986, 95% CI: −1.797 to −0.174), and lower incidence of vomiting (RR = 0.083, 95% CI: 0.026–0.266, *I* ^2^ = 0%) compared to oral chloral hydrate [61].

A 2020 meta‐analysis of 15 RCTs involving 2128 children demonstrated intranasal dexmedetomidine provided superior sedation success rates (RR = 1.14, 95% CI: 1.05–1.25, *I* ^2^ = 79%, *p* = 0.003), lower incidence of vomiting (RR = 0.07, 95% CI: 0.03–0.17, *I* ^2^ = 0%) and coughing (RR = 0.15, 95% CI: 0.05–0.44, *I* ^2^ = 0%) compared to chloral hydrate. Chloral hydrate was associated with longer onset time (MD = −3.54, 95% CI: −5.94 to −1.15, *I* ^2^ = 95%) and recovery time (MD = −30.08, 95% CI: −46.77 to −13.39, *I* ^2^ = 99%) [81].

A 2022 systematic review of7 RCTs involving 1846 children found that for CT and MRIl sedation, intranasal dexmedetomidine (2 μg/kg) is associated with higher sedation success rate (85.07% vs. 71.31%, RR = 1.14, 95% CI: 1.03–1.26, *I* ^2^ = 67.5%) and significantly lower incidence of nausea/vomiting (RR = 0.09, 95% CI: 0.04–0.23, *I* ^2^ = 37.8%) compared to oral chloral hydrate (50–60 mg/kg) [82].

A 2020 meta‐analysis of 34 RCTs involving 2281 children demonstrated that dexmedetomidine (administered intravenously, intranasally, or orally) achieved significantly better sedation than midazolam for parental separation (RR = 0.31, 95% CI: 0.24–0.41, *I* ^2^ = 76%) [70]. A 2023 meta‐analysis of 11 RCTs involving 824 children further showed that intranasal dexmedetomidine outperformed oral midazolam in parental separation (RR = 1.37, 95% CI: 1.14–1.64, *I* ^2^ = 79.3%) and anesthesia induction (RR = 2.08, 95% CI: 1.03–4.22, *I* ^2^ = 81.7%, no publication bias) [71].

A 2023 meta‐analysis of 10 RCTs involving 727 children found intranasal administration of 2–3 mg/kg ketamine had a lower sedation success rate compared to 2–3 μg/kg intranasal dexmedetomidine (46.36% vs. 63.64%, RR = 0.77, 95% CI: 0.63–0.94, *p* = 0.009, *I* ^2^ = 0%) in procedural sedation and premedication [83].

A 2019 meta‐analysis of 6 RCTs involving 412 children found there was no significant difference in sedation failure rates between intravenous dexmedetomidine (1–2 μg/kg bolus +0.5–2 μg/kg/h maintenance) and intravenous propofol (2–3 mg/kg bolus +100–200 μg/kg/min maintenance) (RR = 0.74, 95% CI: 0.09–6.38, *p* = 0.78, *I* ^2^ = 75%) for MRI examinations. Dexmedetomidine was associated with a lower incidence of hypoxemia (RR = 0.11, 95% CI: 0.02–0.55, *p* < 0.01, *I* ^2^ = 0%) [84].

##### 3.6.3.3 Sevoflurane

A 2022 meta‐analysis of 67 RCTs involving 22 380 children reported that for MRI examinations in children aged 0–8 years, the sedation success rates of inhaled sevoflurane (98%, 95% CI: 0.97–0.99) were higher than oral chloral hydrate (94%, 95% CI: 0.91–0.96), oral chloral hydrate plus intranasal dexmedetomidine (95%, 95% CI: 0.92–0.97), midazolam (rectal/oral/intranasal) (36%, 95% CI: 0.14–0.65), intranasal dexmedetomidine (62%, 95% CI: 0.38–0.82), and intranasal dexmedetomidine plus midazolam (94%, 95% CI: 0.78–0.99) [85].

A 2008 RCT including 88 children aged 2–6 years for MRI sedation found that compared with propofol infusion, inhaled sevoflurane was associated with significantly lower sedation failure rate (0% vs. 11%, *p* < 0.05), shorter onset time (3.3 min vs. 4.8 min, *p* < 0.05), and shorter recovery time (3.9 min vs. 8.8 min, *p* < 0.05) [86].

##### 3.6.3.4 Combination of Sedatives

One meta‐analysis and one RCT showed superior success rates of combination regimens compared with a single sedative. Two retrospective studies reported lower adverse events with combination therapy of propofol and midazolam or dexmedetomidine compared to propofol alone.

A 2023 meta‐analysis of 10 RCTs involving 727 children found that 2–3 mg/kg intranasal ketamine alone showed a lower success rate compared to the combination of intranasal ketamine 1–1.5 mg/kg and intranasal dexmedetomidine 1–1.5 μg/kg (33.82% vs. 68.11%, RR = 0.50, 95% CI: 0.27–0.92) for pediatric premedication and procedural sedation [83].

A 2024 RCT including 105 children demonstrated that in CT sedation for cleft lip/palate patients, intranasal dexmedetomidine 2 μg/kg and midazolam 0.05 mg/kg expressed a higher success rate than intranasal dexmedetomidine 2 μg/kg alone (95.9% vs. 80.4%, *p* = 0.016) [87].

A 2017 retrospective study including 897 children for MRI sedation showed propofol plus midazolam reduced risk of airway adverse events compared with propofol alone (2.8% vs. 7.0%, *p* = 0.002) [78].

A 2017 retrospective study including 256 children for MRI sedation found propofol plus dexmedetomidine had lower overall adverse events (5.9% vs. 26.4%, *p* < 0.001) and lower airway obstruction (4.1% vs. 16.1%, *p* = 0.01) when compared with propofol alone [77].

### 3.7 Clinical Question 7: What Medications Should Be Selected as Rescue After Failed Sedation in Pediatric Patients?

#### 3.7.1 Recommendation

7.1 It is recommended dexmedetomidine and sevoflurane as rescue intervention for failed sedation (Recommendation level: A, Level of evidence: 1b, Consensus rate 81.2% (13/16)).
7.2 It is recommended propofol as a rescue intervention for failed sedation (Recommendation level: D, Level of evidence: 5, Consensus rate 93.8% (15/16)).

#### 3.7.2 Rationale for Recommendation

Sedation is considered a failure when a single sedative administration fails to achieve adequate sedation depth, or the patient awakens or exhibits movement during the examination, which leads to incomplete examination [88, 89]. Rescue measures should be implemented to ensure the examination is completed under appropriate sedation depth.

Ideal rescue sedatives should have the following characteristics: rapid onset, high rescue success rates, and minimal additional adverse effects. Additionally, the type, dosage, timing of initial medication administration, and number of rescue attempts should also be considered. Current evidence regarding rescue strategies primarily focuses on failed oral chloral hydrate sedation, while more evidence is needed to support rescue approaches following failure of other sedatives, particularly dexmedetomidine.

A survey from China showed that propofol and inhaled anesthetics are the most commonly used rescue sedatives for children aged 0–1 years, while propofol and dexmedetomidine are the most frequently employed rescue medications for children > 4 years [1]. Non‐intravenous routes are typically preferred for patients without established IV access. Oral rescue medications require the child to be awake and capable of active swallowing, making them unsuitable for patients already in mild or moderate sedation. The “Guidelines for Procedural Sedation by Non‐Anesthesiologists” from the United States noted significant interindividual variability in oral drug absorption and recommended against repeated use of oral medications as rescue measures [5]. Intranasal dexmedetomidine administration is simple, minimally irritating, and easily performed on awake or sedated children. A 2 μg/kg intranasal dexmedetomidine dose showed higher rescue success rates compared to 1–1.5 μg/kg doses, better meeting clinical requirements [90].

Propofol and sevoflurane at anesthetic doses can rapidly achieve deep sedation or even anesthesia. Although direct evidence for propofol as a rescue medication for failed sedation is currently lacking, clinical studies and practice consistently show that intravenous propofol is the most commonly used rescue measure for refractory sedation failure, particularly when initial rescue attempts fail to permit examination completion [1, 91, 92]. Concurrently, studies suggest that inhaled sevoflurane may serve as a rescue option when anesthesia machines and vaporizers are available.

#### 3.7.3 Evidence Summary

There are 4 RCTs showing that intranasal dexmedetomidine 2 μ g/kg achieves higher rescue success rates compared to oral chloral hydrate or intranasal dexmedetomidine 1 μ g/kg.

A 2016 RCT included 156 children aged 1–3 years undergoing MRI who failed to sedate with oral chloral hydrate reported the rescue success rates of adding oral chloral hydrate 25 mg/kg, intranasal dexmedetomidine 1 μ g/kg or 2 μ g/kg were 74.4%, 80.0%, and 94.0%, respectively (*p* < 0.05), and there was no statistical difference in onset time between the 3 groups [93]. Chloral hydrate showed significantly longer recovery time than dexmedetomidine (*p* < 0.05), and dexmedetomidine 2 μg/kg had longer recovery than 1 μg/kg (*p* < 0.05). Another 2016 RCT of 60 children aged 5–36 months undergoing ophthalmic examinations who had failed sedation with chloral hydrate, showed the rescue success rates of intranasal dexmedetomidine 1 and 2 μg/kg were 66.7% and 93.3%, respectively (*p* = 0.021), and there was no significant difference in onset time and recovery time [94]. A 2014 RCT of 213 children aged 1 month–10 years requiring sedation rescue after chloral hydrate failure reported the rescue success rates of adding intranasal dexmedetomidine 1, 1.5 and 2 μg/kg were 83.6%, 89.2%, and 96.2%, respectively [90]. A 2016 RCT of 150 infants aged 1–6 months undergoing MRI after failed chloral hydrate sedation demonstrated the success rates of adding oral chloral hydrate 25 mg/kg, dexmedetomidine 1 and 2 μg/kg sedation were 80%, 94%, and 98%, respectively. No obvious adverse hemodynamic or hypoxemic effects were observed [95].

A 2021 RCT of 336 children requiring rescue after intranasal dexmedetomidine sedation failure during MRI sedation showed that the rescue success rate of 0.4% sevoflurane inhalation was higher than that of 2 mg/kg intranasal ketamine (95.2% vs. 82.1%, RR = 1.159, 95% CI: 1.07, 1.25). The onset time of sevoflurane rescue (5.7 ± 0.5 vs. 10.9 ± 2.7 min, *p* < 0.001) and recovery time (27.4 ± 6.3 vs. 53.8 ± 15.2 min, *p* < 0.001) were shorter than 2 mg/kg intranasal ketamine [88].

### 3.8 Clinical Question 8: Can Off‐Label Medications Be Used for Pediatric Sedation?

#### 3.8.1 Recommendation

8.1 For pediatric sedation, a thorough individualized risk assessment is essential, particularly when utilizing medications such as propofol, remimazolam, ciprofol, dexmedetomidine, esketamine, etomidate, or midazolam. Given the potential for off‐label use and associated safety concerns, administration should be conducted under the direct supervision of a qualified anesthesiologist when clinically warranted (Recommendation Level: B; Level of evidence: 2b, Consensus rate 93.8% (15/16)).

#### 3.8.2 Rationale for Recommendation

Common pediatric sedative agents including propofol, remimazolam, ciprofol, dexmedetomidine, esketamine, etomidate, and midazolam are frequently used off‐label in children.

The package insert for propofol medium/long‐chain triglyceride emulsion injection indicates its approved use for induction and maintenance of general anesthesia, as well as procedural sedation in children older than 1 month. However, when propofol is used for endotracheal intubation in neonates, the sedative effects and circulatory risks are unpredictable, and there is a potential risk of intracranial hemorrhage. Therefore, caution should be exercised during administration of this medicine. Pediatric use of remimazolam tosylate for injection and esketamine hydrochloride injection is unspecified in its labeling. However, RCTs have demonstrated that both remimazolam and esketamine exhibit a good sedative effect and favorable safety profiles when used off‐label for pediatric sedation. Dexmedetomidine hydrochloride injection is not recommended for patients under 18 years per its label and only dexmedetomidine nasal spray is approved for preoperative sedation and anxiolysis in children aged 2 to 6 years. However, dexmedetomidine has been widely used off‐label for procedural sedation, preoperative sedation, and ICU sedation in children aged 1 month to 18 years, demonstrating high success rates with minimal respiratory depression and hemodynamic effects. Etomidate emulsion injection is contraindicated in neonates and infants younger than 6 months according to its labeling. However, studies found single‐dose administration in this population may not significantly impact circulation or cerebral perfusion. Midazolam injection is approved for preoperative sedation, procedural sedation, and ICU sedation in children except neonates. Rapid IV administration in neonates or preterm infants may cause severe hypotension, seizures, and prolonged respiratory depression. Therefore, caution should be exercised when used in this population.

#### 3.8.3 Evidence Summary

An RCT involving 173 neonates demonstrated there was no significant difference in the incidence of decreased oxygen saturation between the group receiving propofol and the group receiving atracurium and sufentanil for non‐emergency endotracheal intubation in neonates (59.6% vs. 65.9%, RD = −6.4, 95% CI: −21 to 8.1, *p* = 0.38). The propofol group exhibited a lower proportion of highly satisfactory sedation (51.7% vs. 92.6%, *p* < 0.001), faster recovery times for respiration (14 min (Interquartile Range, IQR: 8–34 min) vs. 33 min (IQR: 15–56 min), *p* = 0.002) and limb movement (18 min (IQR: 10–43 min) vs. 36 min (IQR: 19–65 min), *p* = 0.003), and a greater decrease in mean arterial pressure compared to baseline (*p* = 0.009) [96]. Cranial ultrasound scans revealed similar worsening intracranial hemorrhage between the two groups (20.6% vs. 17.6%, adjusted RD = 1.2, 95% CI: −13.1 to 15.5, *p* = 0.87).

An effective dose‐finding study including 29 children found that intranasal remimazolam is feasible for preoperative sedation and no adverse effects were observed except for one child who developed excitement [97]. An RCT of 90 children found that intranasal remimazolam premedication achieved mild sedation in 80% of children at 10 min, with 3.3% progressing to deep sedation (OR = 11.90; 95% CI: 3.46–40.92) but caused substantial nasal irritation [98]. These children also exhibited better parental separation (*p* = 0.008) and mask acceptance (*p* = 0.014) compared to the control group. An RCT of 30 pediatric patients receiving intranasal esketamine or intranasal dexmedetomidine for procedural sedation showed no severe adverse events during sedation [99]. An RCT involving 198 pediatric patients undergoing tonsillectomy and adenoidectomy demonstrated that preoperative sedation with intranasal co‐administration of dexmedetomidine and esketamine provided significantly deeper sedation (*p* < 0.001), better separation from parents (*p* = 0.012) and acceptance of the mask for induction (*p* = 0.004), significantly reduced emergence time (*p* < 0.001) and the incidence of postoperative delirium (RR = 0.2595%; 95% CI: 0.11–0.57) than dexmedetomidine alone without significant adverse effects [100].

A recent RCT of 121 children aged 1–3 years undergoing transthoracic echocardiography showed that 0.5–1.5 mg/kg esketamine combined with 1 μg/kg dexmedetomidine did not cause significant changes in HR or SpO_2_ from base line [23]. No serious adverse effects were observed and only four children experienced a transient desaturation not requiring intervention. In an RCT of 111 children undergoing MRI sedation, esketamine‐propofol sedation resulted in a lower dose of propofol for titration (difference in medians(MD) = −64.3 μg/kg/min, 95% CI: −75.9 to 51.9), a shorter emergence time (MD = −9.4 min, 95% CI: −11.4 to 7.4), and a shorter discharge time (MD = −10.1 min, 95% CI: −12.1 to −8.2) without increasing adverse events compared to the combination of dexmedetomidine and propofol [101]. An RCT of 61 pediatric patients showed that remimazolam at 0.5 mg/kg for sedation shortened recovery time compared to esketamine (*p* < 0.05) and did not significantly alter MAP, SpO_2_, or HR (*p* > 0.05) [102]. No adverse events related to remimazolam and esketamine were reported in these trials.

A retrospective cohort study of children undergoing tonsillectomy and adenoidectomy found that, compared to propofol, ciprofol induction resulted in similar hemodynamic trends but higher systolic and diastolic blood pressures at equivalent time points post‐administration (*p* = 0.037 and *p* = 0.012). The ciprofol group also had significantly lower PAED scores at 10 and 20 min post‐extubation (*p* < 0.001 and *p* = 0.046) [103]. Another RCT on the optimal ciprofol induction dose in children showed that 0.6 mg/kg ciprofol led to a lower incidence of poor intubation conditions (8.2%, *p* < 0.0167) [104].

A survey of 791 anesthesiologists from four international anesthesia societies found that 70% used dexmedetomidine for pediatric procedural sedation (68%), preoperative sedation (46%), and ICU sedation (46%) [105]. A systematic review of 9 RCTs with 1076 pediatric patients showed dexmedetomidine had a higher success rate for procedural sedation (OR = 2.90, 95% CI: 1.39–6.07) and lower incidence of respiratory depression (OR = 0.29, 95% CI: 0.15–0.57) compared to other sedatives including chloral hydrate, midazolam, pentobarbital [62]. Another review of 33 studies (31 RCTs and 2 case–control) with 3395 pediatric patients confirmed dexmedetomidine reduced postoperative delirium (OR = 0.23, 95% CI: 0.19–0.27) [106]. Another review of 24 RCTs with 1042 pediatric patients found dexmedetomidine reduced junctional ectopic tachycardia (OR = 0.32, 95% CI: 0.20) and maintained hemodynamic stability (SBP: SMD = −0.35, 95% CI: −0.72 to 0.01; MAP: SMD = −0.83, 95% CI: −1.87 to 0.21; DBP: SMD = −0.79, 95% CI: −1.66 to 0.08; HR: SMD = −1.71, 95% CI: −2.29 to 1.13) [107].

A cohort study involving 50 infants aged 0–11 months demonstrated that a single injection of 0.4 mg/kg etomidate during induction did not impair global or regional cerebral perfusion in neonates or infants with congenital heart disease. The mean arterial blood pressure, cardiac index, and regional cerebral oxygen saturation remained stable above predefined limits [108].

A retrospective study found the success rate of midazolam sedation was 91.67% in 26 neonates without adverse events [109].

### 3.9 Clinical Question 9: What Are the Discharge Criteria for Children After Sedation?

#### 3.9.1 Recommendation

9.1 It is recommended to use tools such as the Aldrete Recovery Score or the Steward Recovery Score to assess recovery in children after sedation. After the children meet the corresponding scoring criteria (e.g., Aldrete score ≥ 9 or Steward score ≥ 5), a comprehensive assessment considering the sedation method, type of examination, individual patient differences, and other recovery indicators is conducted by the sedation provider to determine whether they meet the criteria for safe discharge before being discharged (Recommendation Level: D, Level of evidence: 5, Consensus rate 100% (16/16)).

#### 3.9.2 Rationale for Recommendation

The recommendation for discharge criteria after pediatric sedation is based primarily on the combination of validated assessment tools and clinical indicators. Commonly used tools in clinic include the Aldrete Recovery Score and the Steward Recovery Score. The Aldrete score evaluates dimensions such as motor activity, respiration, circulation, consciousness, and skin color; a score of ≥ 9 indicates the child is close to pre‐sedation status and can be safely discharged. The Steward score focuses on consciousness, airway patency, and motor activity; a score of ≥ 5 suggests good recovery. If a child's Steward score is ≤ 5 before sedation, they can be discharged once their post‐sedation Steward score returns to the pre‐sedation level. These tools quantify vital signs, consciousness level, and airway patency, providing a scientific basis for safe discharge. In multiple clinical studies, Aldrete score ≥ 9 or Steward score ≥ 5 have been used as discharge criteria for pediatric sedation or anesthesia [91, 110, 111, 112]. Clinical observations have shown that when children have independent activities such as sitting up and speaking, and their physiological functions are stable, it can significantly reduce the rate of readmission and ensure safety after discharge. In the comprehensive evaluation, sedation methods (such as duration of sedative drug action, last dose time), examination types, and individual differences (such as age, underlying diseases, etc.) should also be considered.

At present, there is no unified standard for the discharge of children after sedation. The expert group concluded that children should have stable physiological functions (including cardiovascular function, respiratory function, and oxygenation status) and intact protective reflexes before discharge. Experts recommend using the Aldrete score (≥ 9) or the Steward score (≥ 5) as the core criteria for discharge after pediatric sedation, supplemented by a comprehensive assessment tailored to local sedation protocols, the type of examination, and the individual patient differences (such as underlying diseases and age‐appropriate autonomous activity ability, etc.) to ensure safe discharge.

#### 3.9.3 Evidence Summary

Currently, there is no direct research evidence on discharge criteria for children after sedation. However, both clinical studies and practice commonly utilize the Aldrete Score or Steward Score and sometimes clinical observation as assessment tools for determining discharge readiness in children following sedation or anesthesia.

In another RCT involving 60 children undergoing MRI sedation with intranasal dexmedetomidine, a modified Aldrete score of ≥ 9 was used as the discharge criterion during the recovery phase [91].

A retrospective cohort study involving 142 children undergoing flexible bronchoscopy who were sedated by different doses of dexmedetomidine combined with sufentanil, discharge criterion was also based on the modified Aldrete score ≥ 9 [111].

In an RCT of 347 children undergoing MRI sedation with a combination of propofol and ketamine or the propofol‐only group, a modified Aldrete score of 10 was used as a criterion of full recovery and discharge [113].

In an RCT involving 21 children undergoing MRI sedation with sevoflurane or thiopental, the discharge criterion also was a modified Aldrete score of 10 [114].

In an RCT involving 70 children aged 2 to 16 years undergoing colonoscopy under sedation, the Steward scoring system was used during the recovery phase, with a score of ≥ 5 considered indicative of full awakening [112].

In an RCT involving 27 children undergoing dental sedation, discharge criteria were based on clinical observation to confirm that the child was awake, able to sit up independently, and had stable vital signs [115].

In an RCT involving 100 children aged 1 to 7 years undergoing bilateral myringotomy, researchers conducted a comprehensive assessment of respiratory function, mental status, and pain control as PACU discharge criteria [116].

### 3.10 Question 10: Is It Necessary for Children to Fast Before and After Sedation?

#### 3.10.1 Recommendation

10.1 It is recommended that children scheduled for elective moderate to deep sedation follow the 1‐4‐6 fasting guideline. If the examination is urgent, the risks and benefits of the patients need to be evaluated according to the actual situation (Recommendation level: B; Evidence level: 2b, Consensus rate 93.8% (15/16)).
10.2 It is recommended that children start to eat with clear liquids as soon as they are awake after the sedation and followed by gradual dietary advancement (Recommendation level: D; Evidence level: 5, Consensus rate 100% (16/16)).

#### 3.10.2 Rationale for Recommendation

Since protective reflexes are somehow kept during sedation in children, the risk of regurgitation and aspiration is relatively low. It is noted that shortening the fasting time can prevent the occurrence of dehydration and hypoglycemia in children [117, 118]. Additionally, due to the urgency of some examinations, children cannot strictly comply with the fasting algorithm. According to the available literature, in most Chinese hospitals, the 2‐4‐6 principle is required, which is 2 h for clear liquids, 4 h for breast milk, and 6 h for formula [2]. However, the fasting algorithm for pediatric sedation is still in debate worldwide. The international multidisciplinary consensus statement on fasting before procedures sedation in adults and children in 2020 proposed that pre‐sedation assessment should conduct risk stratification for each patient based on evidence‐based factors related to the patient's characteristics, comorbidities, the nature of the procedure, and the nature of the expected sedation technique. For children without clear risk of regurgitation and aspiration, the fasting time for clear liquids can be shortened, while for children with a clearly high risk of regurgitation and aspiration, the “2‐4‐6 fasting principle” should be maintained [119]. Moreover, the European guideline in 2022 recommends that the fasting time for clear liquids before sedation can be shortened to 1 h, and the fasting time for solid foods should be 6 h [120].

The patient's gastrointestinal status (such as delayed gastric emptying, pyloric obstruction, etc.) was considered the main risk factor for aspiration during anesthesia [121]. In recent years, some researchers have proposed that the association between the length of the fasting time, the emptying of gastric contents, and regurgitation and aspiration remains to be verified. However, for healthy patients, the incidence of regurgitation and aspiration is relatively low. Based on the low incidence of regurgitation and aspiration and the extremely low mortality rate during pediatric sedation, it is recommended that children scheduled for procedural sedation follow the 1‐4‐6 fasting algorithm. If the scheduled examination is urgent, a decision can be made based on an assessment of the patient's risks and benefits according to the actual situation.

To date, no research has been available on the intake time after pediatric sedation. The expert consensus on enhanced recovery after surgery in China recommends that children undergoing non‐enteric surgery can eat as soon as they wake up [122]. Based on clinical experience, it is recommended that children can attempt oral intake immediately as soon as they wake up, starting with clear liquids and gradually advancing the diet.

#### 3.10.3 Evidence Summary

A cross‐sectional study involving 114 hospitals showed that most hospitals follow the 2‐4‐6 fasting algorithm, that is 2 h of fasting for liquids, 4 h for breast milk, and 6 h for formula or solids, and no reports of serious regurgitation and aspiration events were reported [123].

An RCT including 131 healthy children aged 1–16 years showed that shortening the fasting time of clear fluids from 2 to 1 h did not affect the incidence of adverse events [124]. The study also showed there were no significant differences between the clear liquid fasting duration of 1 and 2 h in terms of gastric residual volume (Median (Interquartile Range) 0.43 (0.21–0.84) vs. 0.46 (0.19–0.79) mL/kg; *p* = 0.43) and pH value (1.43 (1.30–1.56) vs. 1.44 (1.29–1.68), *p* = 0.66) [124]. The parents' satisfaction was higher when their children fasted from clear fluids for 1 h.

A prospective study including 12 093 children found that the incidences of regurgitation and aspiration in children with a clear liquid fasting time of < 1, 1–2, 2–4, and > 4 h were 0.64%, 0.24%, 0.37%, and 0.36%, respectively. Univariate analysis showed that only a clear liquid fasting time of < 1 h was associated with an increased incidence of regurgitation and aspiration (*p* = 0.047). In addition, children aged 1–3 years (OR = 2.7, 95% CI: 1.3%–5.8%, *p* < 0.01) and those undergoing emergency surgery (OR = 2.8, 95% CI: 1.4–5.7, *p* < 0.01) were associated with an increased incidence of regurgitation and aspiration. There was no significant difference in the incidence of regurgitation and aspiration among the subgroups fasting according to the 6/4/0, 6/4/1, and 6/4/2 protocols (all being 0.03%, *p* < 0.0001), without serious consequences [125].

A multi‐center study by PSRC including 139 142 children sedated by propofol found that there was no significant difference in the incidence of aspiration between the children who were nil per os (NPO) or not NPO (0.097‰ vs. 0.079%, *p* = 0.79) [54].

A secondary analysis of the results of a prospective cohort study, which included 6183 children undergoing emergency sedation, showed that shortening the fasting time had no impact on the overall incidence of adverse events (solid food: OR = 1.00, 95% CI: 0.98–1.02, *p* = 0.91; liquid food: OR = 1.00, 95% CI: 0.98–1.02, *p* = 0.97) or the incidence of vomiting (solid food: OR = 1.00, 95% CI = 0.97–1.03, *p* = 0.79; liquid food: OR = 1.00, 95% CI: 0.96–1.03, *p* = 0.81) [126]. Another meta‐analysis involving 8282 patients that used ketamine for sedation also indicated that no regurgitation or aspiration occurred regardless of whether the children followed the 2‐4‐6 fasting algorithm or not [119, 127].

### 3.11 Clinical Question 11: What Monitoring Is Required During Sedation in Children?

#### 3.11.1 Recommendation

11.1 It is recommended that during pediatric procedural sedation, routinely monitor pulse rate (PR) and oxygen saturation (SpO_2_). When feasible, it is suggested to monitor electrocardiogram (ECG), respiratory rate (RR), and blood pressure (BP) as well (Recommendation level: A; Level of Evidence: 5, Consensus rate 93.8% (15/16)).
11.2 It is recommended that for children with developmental abnormalities or those at risk of respiratory compromise, it is recommended to additionally employ end‐tidal carbon dioxide (PetCO_2_) monitoring (Recommendation level: B; Level of Evidence: 2b, Consensus rate 93.8% (15/16)).

#### 3.11.2 Rationale for Recommendation

During pediatric sedation, common complications include respiratory depression, hypoxemia, and hemodynamic instability [128]. In particular, for patients undergoing deep sedation, prolonged sedation, or those at high risk, a decrease in SpO_2_, hypotension, and bradycardia are likely to occur during the process, which may lead to hypoxia or inadequate organ perfusion [129]. Continuous monitoring of PR, ECG, SpO_2_, RR, and BP enables accurate and rapid diagnosis of complications and facilitates timely initiation of appropriate rescue interventions [130]. In the absence of direct studies to assess the level of evidence, the expert group considers that basic vital sign monitoring is crucial for ensuring the safety of children undergoing sedation in clinical practice. For special patients, ECG and BP monitoring can promptly identify changes in circulatory function and aid in the timely management of cardiovascular complications; hence, the recommendation strength has been upgraded to Level A. Multiple guidelines and consensus statements also recommend continuous monitoring of these parameters during pediatric procedural sedation [16, 19, 37, 40].

During the process of procedural sedation, children are at a higher risk of hypoventilation and apnea, particularly when receiving deep sedation [131]. PetCO_2_ monitoring is an important physiological monitoring tool that provides continuous information on respiratory gas exchange. It enables earlier detection of respiratory abnormalities, even when the patient is receiving supplemental oxygen, thus aiding in the timely identification of potential risks such as respiratory depression and airway obstruction [132]. The study has indicated that PetCO_2_ can provide real‐time feedback on the adequacy of alveolar ventilation during spontaneous breathing, allowing for early recognition of respiratory insufficiency in sedated children [133]. The use of PetCO_2_ monitoring during moderate to deep sedation in children plays a crucial role in preventing severe respiratory‐related adverse events and ensuring patient safety [132].

#### 3.11.3 Evidence Summary

Currently, there is no direct evidence to prove that continuous monitoring of PR, SpO_2_, ECG, RR, and BP is mandatory during the sedation process.

A prospective observational study enrolled 150 children aged 1 to 10 years with developmental disabilities who required brain MRI under sedation [134]. During the sedation process, changes in PetCO_2_ values, the occurrence of hypoxemia, and adverse events were recorded to evaluate the effectiveness of PetCO_2_ monitoring in the early identification of hypoxemia. The results showed that 42.6% of the subjects exhibited significant PetCO_2_ abnormalities during sedation. Hypoxemia (SpO_2_ ≤ 93%) occurred in 18% of the subjects. Among the children with hypoxemia, 70.3% had PetCO_2_ abnormalities recorded before changes in the pulse oximeter were observed. The average time difference between the occurrence of PetCO_2_ abnormalities and hypoxemia was 4.38 ± 1.89 min. The sensitivity of PetCO_2_ abnormalities in predicting hypoxemia was 70.3%, with a specificity of 64.3%. An RCT enrolled 154 pediatric emergency patients aged 1 to 20 years who received intravenous sedation. The result showed the children with PetCO_2_ monitoring had a lower incidence of hypoxemia (1.0% vs. 7.1%, *p* = 0.008), lower incidence of persistent hypoventilation (two consecutive abnormal PetCO_2_ values) (1.0% vs. 7.5%, *p* = 0.02), fewer interventions (OR = 0.25, 95% CI: 0.13–0.50), and more timely interventions (concurrent with or within 30 s of hypoventilation) (OR = 2.26, 95% CI: 1.34–3.81), compared to those without PetCO_2_ monitoring [135]. Delayed interventions were highly associated with the occurrence of SpO_2_ < 95% (OR = 5.31, 95% CI: 2.76–10.22) and SpO_2_ < 90% (OR = 6.19, 95% CI: 1.36–28.11).

### 3.12 Clinical Question 12: How to Manage Delayed Recovery After Sedation in Pediatric Patients?

#### 3.12.1 Recommendation

12.1 It is recommended to define delayed recovery after pediatric sedation as a time interval ≥ 2 h from procedure completion to meeting discharge criterion (Recommendation level: D, Level of evidence: 5, Consensus rate 87.5% (14/16)).
12.2 It is recommended to develop individualized sedation protocols based on the child's weight, outpatient status, prior sedation history, and concurrent use of medications with synergistic sedative effects, to prevent delayed recovery after sedation (Recommendation level: B, Level of evidence: 3b, Consensus rate 100% (16/16)).
12.3 It is recommended that, for pediatric patients with delayed recovery after sedation, prioritize assessment and management of potential adverse events, along with continuous monitoring of vital signs (Recommendation level: D, Level of evidence: 5, Consensus rate 93.8% (15/16)).

#### 3.12.2 Rationale for Recommendation

The definition of delayed recovery after pediatric sedation remains unclear. Some studies define it as the duration between the time from successful sedation to meeting discharge criteria ≥ 2 h. Others define it as the time interval from procedure completion to recovery of consciousness ≥ 2 h [136]. A standardized definition is critical for ensuring homogeneity across clinic practice and research. Based on expert consensus, it is recommended that delayed recovery after pediatric sedation be defined as the duration from procedure completion to meeting discharge criteria ≥ 2 h, given the varying sedation duration and sedation depth for different examinations.

Two retrospective case–control studies identified higher body weight, outpatients, patients with sedation history, patients receiving chloral hydrate, and polypharmacy with antiepileptic drugs as risk factors for delayed recovery [137, 138]. Three RCTs and one systematic review found that prolonged recovery time was associated with polypharmacy [89], chloral hydrate [139], or dexmedetomidine use [140, 141]. Therefore, it is recommended to implement individualized adjustments to the sedation protocol for children with risk factors for delayed recovery.

Currently, there is no established protocol for managing delayed recovery in children after sedation. Monitoring and intervention are required for adverse events occurring during the recovery phase, which may prolong post‐sedation phase [142]. Three retrospective studies identified major adverse events during sedation recovery phase, including hypotension, bradycardia, nausea/vomiting, and airway‐related complications [50, 51, 142]. Based on clinical experience, it is recommended to address adverse events with appropriate interventions and extend continuous monitoring primarily and administer specific antidotes if sedative accumulation is suspected. Determine discharge or admission based on clinical status until discharge criteria are met.

#### 3.12.3 Evidence Summary

Currently, there is no direct evidence regarding the management of delayed recovery after sedation. However, when preventing and addressing delayed recovery, it is essential to consider risk factors associated with it and implement appropriate countermeasures.

Currently, there is no unified definition of delayed recovery after pediatric sedation. Three retrospective case–control/cohort studies defined delayed recovery as a sedation duration (time from successful sedation to meeting discharge criteria) ≥ 2 h [50, 51, 137].

A retrospective case–control study involving 30 003 sedated children reported a delayed recovery rate of 2.8%. Risk factors for delayed recovery included higher body weight (OR = 1.03, 95% CI: 1.01–1.05), outpatients (OR = 1.31, 95% CI: 1.07–1.59), patients with sedation history (OR = 1.25, 95% CI: 1.07–1.44), and patients who received chloral hydrate (OR = 1.47, 95% CI: 1.06–2.03) [137]. Another single‐center retrospective cohort study of 3149 sedated children undergoing MRI or CT scans demonstrated that intranasal dexmedetomidine (2 mcg/kg) combined with oral midazolam (0.5 mg/kg) achieved a median sedation duration of 104 min (IQR: 85–120 min), with a delayed recovery rate of 18.7%. Infants exhibited a longer median sedation time of 110 min (IQR: 90–135 min) and a higher delayed recovery rate of 27.5% [50]. A retrospective case–control study of 1346 children undergoing EEG sedation, the risk factors of delayed recovery included age < 1 year, sedation times ≥ 3, and multiple use of anti‐epileptic drugs [138].

While multiple RCTs have identified delayed recovery times associated with combined sedative regimens [89], use of chloral hydrate [139], or dexmedetomidine [140, 141]. An RCT involving 105 children aged 1–36 months undergoing CT sedation showed that intranasal dexmedetomidine combined with midazolam resulted in longer recovery times compared to dexmedetomidine alone (43 [30–59.5] min vs. 31.5 [20.5–53.5] min, *p* = 0.006) [89]. Another RCT of 100 children aged 1–5 years undergoing CT sedation reported that rectal chloral hydrate administration led to a recovery time of (77.1 ± 19.1) minutes, significantly longer than intranasal dexmedetomidine alone (57.0 ± 21.7 min, *p* < 0.001) or intranasal dexmedetomidine combined with sevoflurane inhalation (60.0 ± 23.9 min, *p* < 0.001) [139]. A systematic review of 5 RCTs involving 405 sedated children found significant delayed recovery with dexmedetomidine compared to other sedatives (WMD = 5.22 min, 95% CI: 0.35–10.09, *p* = 0.04, *I* ^2^ = 92%). Subgroup analysis comparing dexmedetomidine with propofol revealed significant delays (WMD = 10.10 min, 95% CI: 2.93–17.27, *p* = 0.006, *I* ^2^ = 79%) [141]. Additionally, an RCT of 70 children aged 1–7 years undergoing MRI sedation demonstrated that intranasal midazolam combined with intravenous dexmedetomidine prolonged recovery time compared to midazolam combined with intravenous propofol (68.9 ± 31.5 min vs. 40.1 ± 23.0 min) [140].

An observational case–control study of 1590 pediatric patients undergoing MRI sedation reported 5.7% hypotension during recovery following dexmedetomidine sedation. The children with hypotension had 18‐min longer sedation duration (1.5 h vs. 1.2 h, *p* = 0.008) and 30‐min extended recovery time (1.7 h vs. 1.2 h, *p* < 0.001) compared to non‐hypotensive children [142].

A retrospective cohort study of 3149 sedated children identified the most common adverse events as bradycardia after sedation (2.03%), followed by nausea/vomiting (0.98%) and hypotension (0.89%). Another single‐center retrospective study of 17 948 sedated children reported the most frequent adverse events as nausea/vomiting (0.3%), SpO_2_ reduction (0.22%), and upper airway obstructions (0.11%) [51].

## 4 Conclusion

This guideline provided recommendations on 12 clinical issues of pediatric sedation, covering pre‐sedation preparation, practitioner competency, facility requirements, sedation implementation, and recovery management. We emphasized thorough pre‐sedation assessment and preparation, ensuring that staffing and facilities are prepared to handle emergent critical situations, selecting sedation protocols that balance safety, efficacy, and practicality, continuous respiratory and circulatory monitoring throughout sedation and recovery, and discharging the child only after meeting safety criteria. It is hoped that this guideline will contribute to enhancing the safety and efficacy of pediatric sedation for diagnostic examination.

## Funding

This work was supported by the Bethune Charitable Foundation.

## Conflicts of Interest

The authors declare no conflicts of interest.

## Supporting information


**Supplementary Document S1.** Members of the working group and the assignments.

**Supplementary Document S2.** Collection and determination of clinical questions.

**Supplementary Document S3.** Query for children and sedation.

**Supplementary Document S4.** Process of recommendation formulation.

## Data Availability

The data that support the findings of this study are available from the corresponding author upon reasonable request.
