# Supplementary material for: Chinese Society of Pediatric Anesthesiology Guideline for Pediatric Sedation (2025)
Source: Paediatr Anaesth. 2026 Apr 10;36(7):850–71. doi: 10.1002/pan.70178 (PMC13247630; doi:10.1002/pan.70178)
Supplement: Supplementary file 1 — Supplementary Document S1. Members of the working group and the assignments. [file PAN-36-850-s002.docx]

Members of the working group and the Assignments

1. **The Chief Clinical Expert:** oversees decision-making, final guideline drafting, and clinical applicability, and the Chief Methodologist leads top-level design, methodological guidance, training, quality control, and rigor assurance throughout the process.

Chief Clinical Expert:

Xingrong Song(The President of Pediatric Anesthesiology Group of the Chinese Society of Anesthesiology; The Director of Department of Anesthesiology and Perioperative Medicine, , Guangzhou Women and Children’s Medical Center, Guangzhou Medical University)

Chief Methodology Expert:

Yaolong Chen(Director of the Innovation Unit for Evidence Evaluation and Guideline Research at the Chinese Academy of Medical Sciences; Director of the WHO Collaborating Centre for Guideline Implementation and Knowledge Translation )

1. **The Steering Committee:** composed of 5 anesthesiology experts, is responsible for establishing other guideline working groups, managing conflicts of interest, approving the guideline protocol, overseeing the development process, reviewing and finalizing the full guideline, as well as providing necessary consultation and guidance throughout the guideline formulation.

Weifeng Yu (President of the Chinese Society of Anesthesiology, First Affiliated Hospital of Wenzhou Medical University)

Jun Li (Department of Anesthesia and Perioperative Medicine, The Second Affiliated Hospital and Yuying Children's Hospital of Wenzhou Medical University)

Mazhong Zhang (Department of Anesthesiology, Shanghai Children's Medical Center Affiliated to Shanghai Jiaotong University School of Medicine)

Li Zhang (Department of Anesthesiology, Children's Hospital of Nanjing Medical University)

Shuangquan Qu (The Affiliated Children's Hospital Of Xiangya School of Medicine, Central South University, (Hunan Children's Hospital))

1. **The Consensus Expert Pane**l: comprising 16 pediatric anesthesiology experts from Asia, Africa, Europe and Americas, responsible for defining clinical questions, establishing recommendation consensus through the Delphi method, and finalizing guideline content.

Zhen Du (Department of Anesthesiology, The Affiliated Children's Hospital Of Xiangya School of Medicine, Central South University, (Hunan Children's Hospital))

Jianmin Zhang (Department of Anesthesiology, National Center for Children's Health, China Beijing Children's Hospital, Capital Medical University‌)

Ying Xu (Department of Anesthesiology, Children's Hospital of Chongqing Medical University, National Clinical Research Center for Child Health and Disorders, Ministry of Education Key Laboratory of Child Development)

Jijian Zheng (Department of Anesthesiology, Shanghai Children's Medical Center Affiliated to Shanghai Jiaotong University School of Medicine)

Yu Cui (Department of Anesthesiology, Chengdu Women's and Children's Central Hospital)

Hang Tian (Department of Anesthesiology and Perioperative Medicine, Guangzhou Women and Children’s Medical Center, Guangzhou Medical University),

Yunxia Zuo (Past President of the Asian Society for Paediatric Anaesthesiology, Department of Anesthesiology, West China Hospital, Sichuan University)

Lifang Yang (Department of Anesthesiology, Affiliated Children's Hospital of Xi'an Jiaotong University)

Liang Zhong (Department of Anesthesiology, Wuhan Children's Hospital (Wuhan Maternal and Child Healthcare Hospital)Tongji Medical College, Huazhong University of Science & Technology),

Yingping Jia (Department of Anesthesiology, Children's Hospital Affiliated Of Zhengzhou University),

Yue Jin (Department of Anesthesiology, Children's Hospital, Zhejiang University School of Medicine)

John Zhong(Department of Anesthesiology, Oklahoma Children's Hospital)

Maryrose Osazuwa (Department of Anesthesiology, National Hospital Abuja)

Laszlo Vutskits (Division of Anesthesiology, Department of Acute Care Medicine, University Hospitals of Geneva)

Elizabeth Drum (Department of Anesthesiology and Critical Care Medicine, Perelman School of Medicine, Children's Hospital of Philadelphia, University of Pennsylvania)

C Dean Kurth (Department of Anesthesiology and Critical Care Medicine, Children's Hospital of Philadelphia, Perelman School of Medicine at the University of Pennsylvania)

1. **The Evidence Evaluation Group:** consisting of 12 evidence-based medicine (EBM)-trained anesthesiologists, responsible for searching, appraising, synthesizing, and grading the evidence, conducting systematic reviews and preparing evidence summaries and recommendation decision tables.

Lei Yang (Department of Anesthesiology, West China Hospital, Sichuan University)

Yue Huang (Department of Anesthesiology, Shanghai Children's Medical Center Affiliated to Shanghai Jiaotong University School of Medicine)

Siwei Wei (Department of Anesthesiology, The Affiliated Children's Hospital Of Xiangya School of Medicine, Central South University, (Hunan Children's Hospital))

Tingting Wang (Department of Anesthesiology, Wuhan Children's Hospital (Wuhan Maternal and Child Healthcare Hospital), Tongji Medical College, Huazhong University of Science & Technology),

Rui Zhou (Department of Anesthesiology, Children's Hospital Affiliated Of Zhengzhou University)

Heqi Liu (Department of Anesthesiology, National Center for Children's Health, China Beijing Children's Hospital, Capital Medical University‌)

Dongxu Lei (Department of Anesthesiology and Perioperative Medicine, , Guangzhou Women and Children’s Medical Center, Guangzhou Medical University),

Zixin Wang (Department of Anesthesiology and Perioperative Medicine, , Guangzhou Women and Children’s Medical Center, Guangzhou Medical University),

Rui Ma (Affiliated Children's Hospital of Xi'an Jiaotong University),

Qinghua Huang (Department of Anesthesiology, Chengdu Women's and Children's Central Hospital)

Wei Liu (Department of Anesthesiology, Children's Hospital of Chongqing Medical University, National Clinical Research Center for Child Health and Disorders, Ministry of Education Key Laboratory of Child Development)

Dongpi Wang (Department of Anesthesiology, Children's Hospital, Zhejiang University School of Medicine)

1. **The Secretariats**, comprising two guideline methodology and EBM-trained anesthesiologists, responsible for coordinating subgroup work, drafting protocol and manuscripts, conducting surveys, organizing consensus meeting, documenting development processes, and submission for publication.

Dongxu Lei & Liumei Chen (Department of Anesthesiology, Guangzhou Women and Children’s Medical Center, Guangzhou Medical University),

1. **The Methodology Group**, consisting of 2 methodologists, responsible for providing comprehensive methodological guidance and recommendations throughout the guideline development process

Xueping Li & Zhenyu Tang (Chongqing Key Laboratory of Child Neurodevelopment and Cognitive Disorders, Ministry of Education Key Laboratory of Child Development and Disorders, National Clinical Research Center for Child Health and Disorders, Children's Hospital of Chongqing Medical University. Chevidence Lab Child & Adolescent Health, Department of Pediatric Research Institute, Children's Hospital of Chongqing Medical University)

1. **The External Review Group**, comprising three independent pediatric anesthesiology experts, responsible for evaluating the final guideline for scientific validity, clarity, and impartiality while providing actionable feedback on risks, issues, and recommendations.

Rong Wei (Department of Anesthesiology, Shanghai Children's Hospital, School of Medicine, Shanghai Jiao Tong University)

Shoudong Pan (Department of Anesthesiology, Capital Center for Children’s Health, Capital Medical University)

Liming Cheng (Department of Anesthesiology and Surgical Intensive Care Unit, Kunming Children's Hospital)
