# Supplementary material for: Chinese Society of Pediatric Anesthesiology Guideline for Pediatric Sedation (2025)
Source: Paediatr Anaesth. 2026 Apr 10;36(7):850–71. doi: 10.1002/pan.70178 (PMC13247630; doi:10.1002/pan.70178)
Supplement: Supplementary file 2 — Supplementary Document S2. Collection and determination of clinical questions. [file PAN-36-850-s004.docx]

**Collection and determination of clinical questions**

Expert interviews were conducted with pediatric anesthesiologists, pediatricians, and pediatric sedation nurses, yielding 17 clinical questions related to pediatric sedation, ultimately12 key clinical questions were identified. Each expert independently gave a score of 1–7 (1: least important, 7: most important) for the clinical questions during the Delphi voting process. Voting was conducted online and anonymously. The secretariat who was not involved in voting aggregated the results. Clinical questions with 6-7 points that received over 75% of the votes were included in the guideline, while those with 1-2 points that exceeded 75% of the votes were excluded. The remaining clinical questions proceeded to the next round of Delphi voting after discussion and revision during the meeting.

| No | Clinic Question | Proportion of 6-7 points in first round of Delphi | Proportion of 6-7 points in second round of Delphi |  |
| --- | --- | --- | --- | --- |
| 1 | What assessments should be performed prior to pediatric sedation? | 100% | / | Included |
| 2 | Should an anesthesiologist be involved in pediatric sedation? | 78.60% | / | Included |
| 3 | What qualifications should a pediatric sedation practitioner possess? | 92.80% | / | Included |
| 4 | What conditions should the pediatric sedation facility meet? | 92.80% | / | Included |
| 5 | What emergency plans should be in place for pediatric sedation? | 92.80% | / | Included |
| 6 | Should fasting be required before and after pediatric sedation? | 71.40% | 86.67% | Included |
| 7 | What monitoring should be implemented during pediatric sedation? | 71.40% | 80% | Included |
| 8 | What sedation regimen should be selected for pediatric sedation? | 85.70% | / | Included |
| 9 | What remedial measures should be taken after failed pediatric sedation? | 92.80% | / | Included |
| 10 | Is continuous oxygen administration necessary during pediatric sedation? | 35.70% | 20% | Excluded |
| 11 | Should intravenous access be established for sedated children? | 35.70% | 26.67% | Excluded |
| 12 | What criteria should be met for discharge after pediatric sedation? | 92.80% | / | Included |
| 13 | How should delayed recovery after pediatric sedation be managed? | 71.40% | 86.67% | Included |
| 14 | How should the quality of pediatric sedation be evaluated? | 35.70% | 26.67% | Excluded |
| 15 | Can off-label drug use be applied in pediatric sedation? | 50% | 80% | Included |
| 16 | Is it necessary to combine non-pharmacological sedation methods for neonatal sedation? | New question | 66.67% | Excluded after discussion |
| 17 | Is sleep deprivation required prior to pediatric sedation? | New question | 20% | Excluded |
