# Supplementary material for: Chinese Society of Pediatric Anesthesiology Guideline for Pediatric Sedation (2025)
Source: Paediatr Anaesth. 2026 Apr 10;36(7):850–71. doi: 10.1002/pan.70178 (PMC13247630; doi:10.1002/pan.70178)
Supplement: Supplementary file 3 — Supplementary Document S3. Query for children and sedation. [file PAN-36-850-s003.docx]

**Query for children**

**Pubmed Query**

"Child"[MeSH Terms] OR "Child, Preschool"[MeSH Terms] OR "Adolescent"[MeSH Terms] OR "Infant"[MeSH Terms] OR "Infant, Newborn"[MeSH Terms] OR "infan*"[Title] OR "newborn*"[Title] OR "new born*"[Title] OR "perinat*"[Title] OR "neonat*"[Title] OR "baby*"[Title] OR "babies"[Title] OR "toddler*"[Title] OR "minor*"[Title] OR "boy"[Title] OR "girl*"[Title] OR "kid"[Title] OR "kids"[Title] OR "child*"[Title] OR "schoolchild*"[Title] OR "adolescen*"[Title] OR "juvenil*"[Title] OR "youth*"[Title] OR "teen*"[Title] OR "pubescen*"[Title] OR "pediatric*"[Title] OR "paediatric*"[Title] OR "peadiatric*"[Title] OR "prematur*"[Title] OR "preterm*"[Title] OR "pre-school"[Title] OR "preschool*"[Title]

**Cochrane Query**

#1. MeSH descriptor child explode all trees OR MeSH descriptor adolescent explode all trees OR MeSH descriptor infant explode all trees

#2. (infan* or newborn* or 'new born*' or perinat* or neonat* or baby* or babies or toddler* or minor* or boy* or girl* or kid or kids or child* or schoolchild* or adolescen* or juvenil* or youth* or teen* or pubescen* or pediatric* or paediatric* or peadiatric* or prematur* or preterm* or 'pre school' or preschool*):ti,ab,kw

#3. (#1 or #2 )

**Embase Query**

1. 'child'/exp OR 'adolescent'/exp OR 'infant'/exp
2. infan*:ab,ti OR newborn*:ab,ti OR 'new born*':ab,ti OR perinat*:ab,ti OR neonat*:ab,ti OR baby*:ab,ti OR babies:ab,ti OR toddler*:ab,ti OR minor*:ab,ti OR boy*:ab,ti OR girl*:ab,ti OR kid:ab,ti OR kids:ab,ti OR child*:ab,ti OR schoolchild*:ab,ti OR adolescen*:ab,ti OR juvenil*:ab,ti OR youth*:ab,ti OR teen*:ab,ti OR pubescen*:ab,ti OR pediatric*:ab,ti OR paediatric*:ab,ti OR peadiatric*:ab,ti OR prematur*:ab,ti OR preterm*:ab,ti OR 'pre school':ab,ti OR preschool*:ab,ti
3. #1 OR #2

**Web of Science Query**

((((((((((((((((((((((((((TS=(infan*)) OR TS=(newborn*)) OR TS=("new born*")) OR TS=(perinat*)) OR TS=(neonat*)) OR TS=(baby*)) OR TS=(babies)) OR TS=(toddler*)) OR TS=(minor*)) OR TS=(boy*)) OR TS=(girl*)) OR TS=(kid)) OR TS=(kids)) OR TS=(child*)) OR TS=(schoolchild*)) OR TS=(adolescen*)) OR TS=(juvenil*)) OR TS=(youth*)) OR TS=(teen*)) OR TS=(pubescen*)) OR TS=(pediatric*)) OR TS=(paediatric*)) OR TS=(peadiatric*)) OR TS=(prematur*)) OR TS=(preterm*)) OR TS=(pre-school)) OR TS=(preschool*)

**CNKI Query**

(((SU=儿童) OR (SU=青少年) OR (SU=婴儿) OR (SU=学龄前) OR (SU=学生) OR (SU=青春期) OR (SU=儿科) OR (SU=男孩) OR (SU=女孩) OR (SU=新生儿))

**CBM Query**

1. (("儿童"[不加权:扩展]) OR "青少年"[不加权:扩展]) OR "婴儿"[不加权:扩展]
2. "儿童"[常用字段:智能] OR "青少年"[常用字段:智能] OR "婴儿"[常用字段:智能] OR "学 龄前"[常用字段:智能] OR "学生"[常用字段:智能] OR "青春期"[常用字段:智能] OR "儿科"[常用字段:智能] OR "男孩"[常用字段:智能] OR "女孩"[常用字段:智能] OR "新生儿"[常用字段:智能]
3. #1 OR #2

**Wanfang Query**

1. 主题:(儿童) or 主题:(青少年) or 主题:(婴儿) or 主题:(学龄前) or 主题:(学生) or 主题:(青春期) or 主题:(儿科) or 主题:(男孩) or 主题:(女孩) or 主题:(新生儿)

**Query for sedation**

**Pubmed Query**

("deep sedation"[MeSH Terms]) OR ("conscious sedation"[MeSH Terms]) OR ("Hypnotics and Sedatives" [MeSH Terms]) OR (procedural sedation [Title/Abstract]) OR (moderate sedation [Title/Abstract]) OR (sedati*[Title/Abstract])

**Cochrane Query**

#1 MeSH descriptor: [Deep Sedation] explode all trees

#2 MeSH descriptor: [Conscious Sedation] explode all trees

#3 MeSH descriptor: [Hypnotics and Sedatives] explode all trees

#4 (moderate sedation):ti,ab,kw OR (procedural sedation):ti,ab,kw OR (sedati*):ti,ab,kw (Word variations have been searched)

#5 #1 OR #2 OR #3 OR #4

**Embase Query**

'sedation'/exp OR 'conscious sedation'/exp OR 'hypnotic sedative agent'/exp OR 'sedative agent'/exp OR 'deep sedation'/exp OR sedati*:ti,ab,kw

**Web of Science Query**

(TS=(deep sedation) OR TS=(Conscious Sedation) OR TS=(moderate sedation) OR TS=(procedural sedation) OR TS=(sedati*))

**CNKI Query**

(SU= 镇静) OR (SU= 镇静效果) OR (SU=镇静剂) OR (SU=镇静作用) OR (SU=镇静药) OR (SU=镇静催眠) OR (SU=过度镇静) OR (SU= 深度镇静) OR (TKA=镇静) OR (SU=中度镇静)

**Wanfang Query**

主题:(镇静) or 主题:(镇静药) or 主题:(深度镇静) or 主题:(中度镇静) or 题名或关键词:(镇静)

**CBM Query**

#1 "催眠药和镇静药"[不加权:扩展] OR "深度镇静"[不加权:扩展] OR "清醒镇静"[不加权:扩展]

#2) "中度镇静"[常用字段:智能] OR "中深度镇静"[常用字段:智能] OR "门诊镇静"[常用字段:智能] OR "镇静"[常用字段:智能] OR "镇静药"[常用字段:智能]

#3) (#2) OR (#1)

****Query for children+sedation****

****Pubmed** Query**

(("deep sedation"[MeSH Terms]) OR ("conscious sedation"[MeSH Terms]) OR ("Hypnotics and Sedatives" [MeSH Terms]) OR (procedural sedation [Title/Abstract]) OR (moderate sedation [Title/Abstract]) OR (sedati*[Title/Abstract])) AND ("Child"[MeSH Terms] OR "Child, Preschool"[MeSH Terms] OR "Adolescent"[MeSH Terms] OR "Infant"[MeSH Terms] OR "Infant, Newborn"[MeSH Terms] OR "infan*"[Title] OR "newborn*"[Title] OR "new born*"[Title] OR "perinat*"[Title] OR "neonat*"[Title] OR "baby*"[Title] OR "babies"[Title] OR "toddler*"[Title] OR "minor*"[Title] OR "boy"[Title] OR "girl*"[Title] OR "kid"[Title] OR "kids"[Title] OR "child*"[Title] OR "schoolchild*"[Title] OR "adolescen*"[Title] OR "juvenil*"[Title] OR "youth*"[Title] OR "teen*"[Title] OR "pubescen*"[Title] OR "pediatric*"[Title] OR "paediatric*"[Title] OR "peadiatric*"[Title] OR "prematur*"[Title] OR "preterm*"[Title] OR "pre-school"[Title] OR "preschool*"[Title]**)**

**Cochrane Query**

**#1 MeSH descriptor: [Deep Sedation] explode all trees**

**#2 MeSH descriptor: [Conscious Sedation] explode all trees**

**#3 MeSH descriptor: [Hypnotics and Sedatives] explode all trees**

**#4 (moderate sedation):ti,ab,kw OR (procedural sedation):ti,ab,kw OR (sedati*):ti,ab,kw (Word variations have been searched)**

**#5 #1 OR #2 OR #3 OR #4**

**#6 MeSH descriptor: [Child] explode all trees**

**#7 MeSH descriptor: [Adolescent] explode all trees**

**#8 MeSH descriptor: [Infant] explode all trees**

**#9 #6 OR #7 OR #8**

**#10 (infan* or newborn* or 'new born*' or perinat* or neonat* or baby* or babies or toddler* or minor* or boy* or girl* or kid or kids or child* or schoolchild* or adolescen* or juvenil* or youth* or teen* or pubescen* or pediatric* or paediatric* or peadiatric* or prematur* or preterm* or 'pre school' or preschool*):ti,ab,kw**

**#11 #9 OR #10**

**#12 # 11 AND #5**

**Embase Query**

#1 'sedation'/exp OR 'conscious sedation'/exp OR 'hypnotic sedative agent'/exp OR 'sedative agent'/exp OR 'deep sedation'/exp OR sedati*:ti,ab,kw

#2 'child'/exp OR 'adolescent'/exp OR 'infant'/exp

#3 infan*:ab,ti OR newborn*:ab,ti OR 'new born*':ab,ti OR perinat*:ab,ti OR neonat*:ab,ti OR baby*:ab,ti OR babies:ab,ti OR toddler*:ab,ti OR minor*:ab,ti OR boy*:ab,ti OR girl*:ab,ti OR kid:ab,ti OR kids:ab,ti OR child*:ab,ti OR schoolchild*:ab,ti OR adolescen*:ab,ti OR juvenil*:ab,ti OR youth*:ab,ti OR teen*:ab,ti OR pubescen*:ab,ti OR pediatric*:ab,ti OR paediatric*:ab,ti OR peadiatric*:ab,ti OR prematur*:ab,ti OR preterm*:ab,ti OR 'pre school':ab,ti OR preschool*:ab,ti

#4 #2 OR #3

#5 #1 AND #4

**Web of Science Query**

(TS=(deep sedation) OR TS=(Conscious Sedation) OR TS=(moderate sedation) OR TS=(procedural sedation) OR TS=(sedati*)) AND (((((((((((((((((((((((((((TS=(infan*)) OR TS=(newborn*)) OR TS=("new born*")) OR TS=(perinat*)) OR TS=(neonat*)) OR TS=(baby*)) OR TS=(babies)) OR TS=(toddler*)) OR TS=(minor*)) OR TS=(boy*)) OR TS=(girl*)) OR TS=(kid)) OR TS=(kids)) OR TS=(child*)) OR TS=(schoolchild*)) OR TS=(adolescen*)) OR TS=(juvenil*)) OR TS=(youth*)) OR TS=(teen*)) OR TS=(pubescen*)) OR TS=(pediatric*)) OR TS=(paediatric*)) OR TS=(peadiatric*)) OR TS=(prematur*)) OR TS=(preterm*)) OR TS=(pre-school)) OR TS=(preschool*))

**CNKI Query**

((SU=儿童) OR (SU=青少年) OR (SU=婴儿) OR (SU=学龄前) OR (SU=学生) OR (SU=青春期) OR (SU=儿科) OR (SU=男孩) OR (SU=女孩) OR (SU=新生儿)) AND ((SU= 镇静) OR (SU= 镇静效果) OR (SU=镇静剂) OR (SU=镇静作用) OR (SU=镇静药) OR (SU=镇静催眠) OR (SU=过度镇静) OR (SU= 深度镇静) OR (SU= 中度镇静) OR (TKA=镇静))

**Wanfang Query**

(主题:(儿童) or 主题:(青少年) or 主题:(婴儿) or 主题:(学龄前) or 主题:(学生) or 主题:(青春期) or 主题:(儿科) or 主题:(男孩) or 主题:(女孩) or 主题:(新生儿))AND(主题:(镇静) or 主题:(镇静药) or 主题:(深度镇静) or 主题:(中度镇静) or题名或关键词:(镇静))

**CBM Query**

#1 "催眠药和镇静药"[不加权:扩展] OR "深度镇静"[不加权:扩展] OR "清醒镇静"[不加权:扩展]

#2) "中度镇静"[常用字段:智能] OR "中深度镇静"[常用字段:智能] OR "门诊镇静"[常用字段:智能] OR "镇静"[常用字段:智能] OR "镇静药"[常用字段:智能]

#3) (#2) OR (#1)

#4) (("儿童"[不加权:扩展]) OR "青少年"[不加权:扩展]) OR "婴儿"[不加权:扩展]

#5) "儿童"[常用字段:智能] OR "青少年"[常用字段:智能] OR "婴儿"[常用字段:智能] OR "学 龄前"[常用字段:智能] OR "学生"[常用字段:智能] OR "青春期"[常用字段:智能] OR "儿科"[常用字段:智能] OR "男孩"[常用字段:智能] OR "女孩"[常用字段:智能] OR "新生儿"[常用字段:智能]

#6) (#5) OR (#4)

#7) (#6) AND (#3)
