# Supplementary material for: Chinese Society of Pediatric Anesthesiology Guideline for Pediatric Sedation (2025)
Source: Paediatr Anaesth. 2026 Apr 10;36(7):850–71. doi: 10.1002/pan.70178 (PMC13247630; doi:10.1002/pan.70178)
Supplement: Supplementary file 4 — Supplementary Document S4. Process of recommendation formulation. [file PAN-36-850-s001.docx]

Process of recommendation formulation

The Evidence Evaluation Group developed evidence recommendation forms and drafted 33 recommendations, while the Consensus Expert Panel conducted two rounds of Delphi voting, and 2 rounds of discussion(online and one offline). Consensus Expert Panel members independently gave a chose of “Agree”, “Disagree” or “Uncertain1” for the recommendation during the Delphi voting process. Voting was conducted online and anonymously. The secretariat who was not involved in voting aggregated the results. The recommendations that received “Agree”over 75% of the votes were included in the guideline, while those with disagree that exceeded 75% of the votes were excluded. The remaining recommendations and those identified as needing modification during the discussion proceeded to the next round of Delphi voting. Through two rounds of Delphi method, the consensus expert group reached a consensus on 31 recommended opinions for 12 clinical questions, and merged some of the recommended opinions, ultimately determining 28 recommended opinions.

**First round of Delphi for Recommendation**

| **Clinical Question 1: What assessments are required prior to pediatric sedation?** | **Agree** | **Disagree** | **Uncertain** | Additional notes |
| --- | --- | --- | --- | --- |
| 1.1 It is recommended to evaluate the child's upper respiratory infection (URI) status prior to procedural sedation. (Recommendation Level: B; Level of Evidence: 2b) | 100.0% | 0.0% | 0.0% |  |
| 1.2 For children undergoing head/neck MRI, airway patency should be assessed before sedation to determine the need for artificial airway support. (Recommendation Level: C; Level of Evidence: 4) | 93.75% | 0.0% | 6.25% |  |
| 1.3 It is recommended to evaluate the child's developmental status prior to MRI sedation. (Recommendation Level: B; Level of Evidence: 2b) | 100.0% | 0.0% | 0.0% |  |
| **Clinical Question 2: Can off-label medications be used for pediatric sedation?** |  |  |  |  |
| **2.1** It is recommended that during pediatric sedation, off-label use of propofol,  remimazolam, ciprofol, dexmedetomidine, esketamine, etomidate, and midazolam be considered based on individual patient conditions, with careful assessment of the risks associated with sedative medications. (Recommendation Level:B; Level of evidence:2b) | 93.75% | 6.25% | 0.0% |  |
| **Clinical Question 3: Should an anesthesiologist be involved in pediatric sedation?** |  |  |  |  |
| 3.1 It is recommended that anesthesiologists be involved in pediatric sedation procedures (Recommendation level: B; Level of evidence: 2b). | 93.75% | 0.0% | 6.25% |  |
| 3.2 For sedation regimens using a single agent in ASA class ≤II children, sedation may be administered by non-anesthesiologists (e.g., trained physicians or nurses)  under appropriate qualifications (Recommendation level: C; Level of evidence: 2b). | 50.0% | 12.50% | 37.50% |  |
| **Clinical Question 4: Which qualifications should pediatric sedation implementers possess?** |  |  |  |  |
| **4.1** Valid certifications for Pediatric Basic Life Support (PBLS) and Pediatric Advanced Life Support (PALS) are recommended for pediatric sedation implementers. (Recommendation level: D, Level of evidence: 5) | 68.75% | 0.0% | 31.25% | Considerting the critical importance of this recommendation, consensus experts suggested upgraded strength of recommendation level for second Delphi |
| **4.2** Qualification of attending physician or above, and over one-year experience in pediatric anesthesia is recommended for pediatric sedation implementers. (Recommendation level: D, Level of evidence: 5) | 68.75% | 6.25% | 25.0% |  |
| **4.3** Structured training in sedation (including assessment of first-aid simulation) is recommended for pediatric sedation implementers (Recommendation level: D, Level of evidence: 5) | 87.5% | 0.0% | 12.5% |  |
| **Clinical Question 5: What conditions should be in place for a pediatric sedation facility?** |  |  |  |  |
| **5.1** Recommendation: All healthcare facilities providing pediatric sedation procedures should be equipped with dedicated sedation rooms and post-sedation recovery rooms. (Recommendation level: D; Level of Evidence: 5) | 100.0% | 0.0% | 0.0% | Considerting the critical importance of this recommendation, consensus experts suggested upgraded strength of recommendation level for second Delphi |
| **5.2** Recommendation: Pediatric sedation facilities should be equipped with basic  monitoring equipment, including pulse oximetry, electrocardiogram (ECG), blood  pressure monitors, capnography, and stethoscopes. (Recommendation level: D; Level of Evidence: 5) | 100.0% | 0.0% | 0.0% |  |
| **5.3** Recommendation: Pediatric sedation facilities should be equipped with emergency equipment, including: Airway management supplies: Oxygen supply devices with flow regulators (at least two independent oxygen sources), anesthesia machines/ventilators, manual resuscitators, nasal cannulas and face masks, oropharyngeal/nasopharyngeal  airways, supraglottic airway devices (e.g., laryngeal masks), laryngoscopes,  endotracheal tubes with stylets, and suction devices; Defibrillators, emergency  medications, and a crash cart with cardiopulmonary resuscitation (CPR) equipment; Communication devices. (Recommendation level: D; Level of Evidence: 5) | 100.0% | 0.0% | 0.0% |  |
| **Clinical Question 6: What contingency plans should be established for pediatric sedation?** |  |  |  |  |
| **6.1** It is recommended that contingency plans for peri-sedation nausea and vomiting be  established regardless of the patient's fasting status, to prevent severe adverse events such as aspiration. (Recommendation level: C, Level of evidence: 4) | 93.75% | 0.0% | 6.25% | Considerting the critical importance of this recommendation, consensus experts suggested upgraded strength of recommendation level for second Delphi |
| **6.2** Contingency plans should be developed for airway impairment or respiratory  suppression (e.g., laryngospasm, endotracheal intubation, cricothyroid membrane  puncture), as well as for hypoxia-induced hypotension, cardiac arrest, and other critical adverse events, particularly in high-risk pediatric patients. (Recommendation level: A, Level of evidence: 1a) | 100.0% | 0.0% | 0.0% |  |
| **6.3** Continuous monitoring of sedation depth is advised, with contingency plans  addressing both inadequate sedation and oversedation. (Recommendation level: B, Level of evidence: 2b) | 100.0% | 0.0% | 0.0% |  |
| **6.4** Standardized contingency plans should be established for sedative overdose caused by multifactorial etiologies. (Recommendation level: C, Level of evidence: 4) | 100.0% | 0.0% | 0.0% | It was included in clinic question 12 and delete this recommendation. |
| **6.5** In settings lacking cardiopulmonary support capabilities, backup emergency plans  should include an activatable emergency medical service (EMS) protocol to address life- threatening complications. (Recommendation level: D, Level of evidence: 5) | 93.75% | 0.0% | 6.25% | It was included in clinic question 5 and delete this recommendation. |
| **6.6** A standardized sedation safety checklist is recommended to enhance protocol  consistency during contingency plan execution. (Recommendation level: D, Level of evidence: 5) | 100.0% | 0.0% | 0.0% | It was included in clinic question 5and 11, delete this recommendation. |
| **Clinical Question 7: What is the optimal sedation regimen for pediatric patients?** |  |  |  |  |
| **7.1** Intranasal dexmedetomidine is recommended as the first-line sedative for pediatric patients, while chloral hydrate is not recommended due to increased risk of distress and adverse effects. (Recommendation level A, Level of evidence: 1a) | 68.75% | 18.75% | 12.5% |  |
| **7.2** Oral or intranasal midazolam is suggested for moderate sedation.  (Recommendation level B, Level of evidence: 1b) | 68.75% | 25.0% | 6.25% |  |
| **7.3** For procedures requiring deep sedation (e.g., MRI), sevoflurane or combination regimens are recommended.(Recommendation level A, Level of evidence: 1a) | 62.5% | 12.5% | 25.0% |  |
| **Clinical Question 8: What medications should be selected as rescue after failed sedation in pediatric patients?** |  |  |  |  |
| **8.1** Intranasal dexmedetomidine (2 µg/kg) is recommended as a rescue intervention for failed oral chloral hydrate sedation.(Recommendation level: A, Level of evidence: 1a) | 68.75% | 6.25% | 25.0% |  |
| **8.2** Sevoflurane is recommended as a rescue intervention for failed intranasal dexmedetomidine sedation.(Recommendation level: A, Level of evidence: 1b) | 75.0% | 6.25% | 18.75% |  |
| **8.3** Propofol is recommended as a rescue intervention for failed sedation (Recommendation level: D, Level of evidence: 5) | 68.75% | 18.75% | 12.5% |  |
| **Clinical Question 9: What are the discharge criteria for pediatric after sedation?** |  |  |  |  |
| **9.1** It is recommended to use assessment tools such as the Aldrete recovery score and the Steward recovery score to evaluate the recovery status of children after sedation. When pediatric patients reach the corresponding score thresholds (e.g., Aldrete score ≥9 or Steward score ≥5), a comprehensive assessment should be conducted by professional medical personnel, taking into account the sedation method, individual variation, and other recovery indicators. Only when the child accord with the safe discharge criteria should discharge be permitted. (Recommendation level: A, Level of evidence: 1b) | 93.75% | 6.25% | 0.0% |  |
| **Question 10:Is it necessary for children to fast before and after sedation?** |  |  |  |  |
| **10.1** Children scheduled for elective moderate to deep sedation follow the 2-4-6 fasting guideline. If the examination is urgent, the risks and benefits of the patients need to be evaluated according to the actual situation to make a decision. (Recommendation level: A; Evidence level: 2b) | 68.75% | 12.5% | 18.75% |  |
| **10.2** It is recommended that children start to eat as soon as they are awake. (Recommendation level:D; Evidence level: 5) | 75.0% | 6.25% | 18.75% |  |
| **Clinical Question 11: What monitoring is required during sedation in children?** |  |  |  |  |
| **11.1** During procedural sedation in children, continuous monitoring of heart rate (HR), electrocardiogram (ECG), oxygen saturation (SpO₂), blood pressure (BP), and respiratory rate (RR) is recommended. (Recommendation Level: D, Level of evidence: 5). | 75.0% | 6.25% | 18.75% |  |
| **11.2** For children undergoing moderate-to-deep sedation, additional monitoring with end-tidal carbon dioxide (EtCO₂) is recommended. (Recommendation Level: A, Level of evidence: 1a). | 87.5% | 6.25% | 6.25% |  |
| **11.3** During procedural sedation in children, monitoring sedation depth using the Bispectral Index (BIS) or Narcotrend Index (NI) is recommended to optimize drug dosing and reduce complications. (Recommendation Level: B, Level of evidence: 2b). | 31.25% | 31.25% | 37.5% |  |
| **Clinical Question 12: How to manage delayed recovery after sedation in pediatric patients?** |  |  |  |  |
| **12.1** It is recommended to define delayed recovery after pediatric sedation as a time interval ≥2 hours from procedure completion to meeting discharge criteria.  (Recommendation level: B, Level of evidence: 3b) | 87.5% | 6.25% | 6.25% | Considerting the critical importance of this recommendation, consensus experts suggested upgraded strength of recommendation level for second Delphi |
| **12.2** It is recommended to develop individualized sedation protocols based on factors  such as the child's weight, outpatient status, prior sedation history, and concurrent use of medications with synergistic sedative effects, to prevent delayed recovery after sedation. (Recommendation level: B, Level of evidence: 3b) | 100.0% | 0.0% | 0.0% |  |
| **12.3** For pediatric patients with delayed recovery after sedation, it is recommended to  prioritize assessment and management of potential adverse events, along with continuous monitoring of vital signs. (Recommendation level: D, Level of evidence: 5) | 93.75% | 0.0% | 6.25% |  |

**Second round of Delphi for Recommendation**

| **Clinical Question 3: Should an anesthesiologist be involved in pediatric sedation?** | **Agree** | **Disagree** | **Uncertain** | Additional notes |
| --- | --- | --- | --- | --- |
| **3.2** For sedation regimens using a single agent (dexmedetomidine or midazolam)in ASA class ≤II children, sedation can be administered by non-anesthesiologists (e.g., trained physicians or nurses) under appropriate qualifications (Recommendation Level C; Level of evidence:4). | 87.50% | 6.25% | 6.25% |  |
| **Clinical Question 4: Which qualifications should pediatric sedation implementers possess?** |  |  |  |  |
| 4.1 Capabilities of Pediatric Basic Life Support (PBLS) for pediatric sedation implementers, and capabilities of Pediatric Advanced Life Support (PALS) in addition for moderate to deep sedation. (Recommendation Level: A，Level of evidence: 5) | 100.00% | 0.00% | 0.00% |  |
| 4.2 Qualification of attending physician and above, or over one-year experience in pediatric anesthesia is recommended for pediatric sedation implementers providing moderate to deep sedation, or for children under 6 years, or for high risk children.(Recommendation Level: A，Level of evidence: 5) | 100.00% | 0.00% | 0.00% |  |
| 4.3 Structured training in sedation(including assessment of first-aid simulation) is recommended for pediatric sedation implementers (Recommendation Level: A，Level of evidence: 5) | 100.00% | 0.00% | 0.00% |  |
| **Clinical Question 5: What conditions should be in place for a pediatric sedation facility?** |  |  |  |  |
| **5.1** Recommendation: All healthcare facilities providing pediatric sedation procedures should be equipped with dedicated sedation rooms and post-sedation recovery rooms. (Recommendation Level: A，Level of evidence: 5) | 100.00% | 0.00% | 0.00% |  |
| **5.2** Recommendation: Pediatric sedation facilities should be equipped with emergency equipment, including:Airway management supplies: Oxygen supply devices with flow regulators (at least two independent oxygen sources), anesthesia machines/ventilators, manual resuscitators, nasal cannulas and face masks, oropharyngeal/nasopharyngeal airways, supraglottic airway devices (e.g., laryngeal masks), laryngoscopes, endotracheal tubes with stylets, and suction devices; Defibrillators, emergency medications, and a crash cart with cardiopulmonary resuscitation (CPR) equipment; Communication devices. (Recommendation Level: A，Level of evidence: 5) | 100.00% | 0.00% | 0.00% |  |
| **5.3** Recommendation: The Pediatric sedation areas should be equipped with basic monitoring devices, including pulse oximetry, electrocardiogram (ECG), blood pressure monitor, and stethoscope; capnography is strongly recommended (Recommendation Level: A，Level of evidence: 5) | 100.00% | 0.00% | 0.00% |  |
| **Clinical Question 7: What is the optimal sedation regimen for pediatric patients?** |  |  |  |  |
| **7.1** Oral midazolam is recommended for pediatric mild sedation. (Recommendation level A, Level of evidence: 1b) | 93.75% | 6.25% | 0.00% |  |
| **7.2** Dexmedetomidine is recommended for pediatric moderate sedation. (Recommendation level A, Level of evidence: 1a) | 87.50% | 6.25% | 6.25% |  |
| **7.3** Sevoflurane or combination regimens are recommended for pediatric deep sedation.(Recommendation level A, Level of evidence: 1a) | 87.50% | 12.50% | 0.00% |  |
| **Clinical Question 8: What medications should be selected as rescue after failed sedation in pediatric patients?** |  |  |  |  |
| **8.1** Dexmedetomidine and sevoflurane are recommended as a rescue intervention for failed sedation.(Recommendation level: A, Level of evidence: 1b) | 81.25% | 0.00% | 18.75% |  |
| **8.2** Propofol is recommended as a rescue intervention for failed sedation (Recommendation level: D, Level of evidence: 5) | 93.75% | 0.00% | 6.25% |  |
| **Clinical Question 9: What are the discharge criteria for pediatric after sedation?** |  |  |  |  |
| **9.1** It is recommended to use tools such as the Aldrete Recovery Score or the Steward Recovery Score to assess recovery in children after sedation. Once the patient meets the respective scoring criteria (e.g., Aldrete score ≥9 or Steward score ≥5), and in consideration of the sedation method, type of procedure, individual patient differences, and other recovery indicators, a comprehensive evaluation should be conducted by qualified healthcare professionals to determine if the child meets the safety criteria for discharge. Only then should the child be discharged. (Recommendation level: D; Level of evidence: 5) | 100.00% | 0.00% | 0.00% |  |
| **Question 10:Is it necessary for children to fast before and after sedation?** |  |  |  |  |
| 10.1 Children scheduled for elective moderate to deep sedation follow the 1-4-6 fasting guideline. If the examination is urgent, the risks and benefits of the patients need to be evaluated according to the actual situation to make a decision.（Recommendation level: B; Level of evidence: 2b） | 93.75% | 0.00% | 6.25% |  |
| 10.2 It is recommended that children start to eat with clear liquidsas as they are awake, followed by gradual dietary advancement.（Recommendation level:D; Level of evidence: 5） | 100.00% | 0.00% | 0.00% |  |
| **Clinical Question 11: What monitoring is required during sedation in children?** |  |  |  |  |
| ****11.1** During pediatric procedural sedation, it is recommended to routinely monitor heart rate (HR) and oxygen saturation (SpO₂). When feasible, it is suggested to monitor electrocardiogram (ECG), respiratory rate (RR), and blood pressure (BP) as well. (Recommendation level: A;** Level of evidence**: 5.)** | 93.75% | 6.25% | 0.00% |  |
| ****11.2** For children with developmental abnormalities or those at risk of respiratory compromise, it is recommended to additionally employ end-tidal carbon dioxide (EtCO₂) monitoring. (**Recommendation level**: B;** Level of evidence**: 2b).** | 93.75% | 6.25% | 0.00% |  |
| **Clinical Question 12: How to manage delayed recovery after sedation in pediatric patients?** |  |  |  |  |
| **12.1** It is recommended to define delayed recovery after pediatric sedation as a time interval ≥2 hours from procedure completion to meeting discharge criteria. (Recommendation level: D, Level of evidence**:** 5) | 87.50% | 12.50% | 0.00% |  |
